# Induction of Covalently Crosslinked p62 Oligomers with Reduced Binding to Polyubiquitinated Proteins by the Autophagy Inhibitor Verteporfin

**DOI:** 10.1371/journal.pone.0114964

**Published:** 2014-12-10

**Authors:** Elizabeth Donohue, Aruna D. Balgi, Masaaki Komatsu, Michel Roberge

**Affiliations:** 1 Department of Biochemistry and Molecular Biology, University of British Columbia, Vancouver, British Columbia, Canada; 2 Tokyo Metropolitan Institute of Medical Science, Tokyo, Japan; IISER-TVM, India

## Abstract

Autophagy is a cellular catabolic process responsible for the degradation of cytoplasmic constituents, including organelles and long-lived proteins, that helps maintain cellular homeostasis and protect against various cellular stresses. Verteporfin is a benzoporphyrin derivative used clinically in photodynamic therapy to treat macular degeneration. Verteporfin was recently found to inhibit autophagosome formation by an unknown mechanism that does not require exposure to light. We report that verteporfin directly targets and modifies p62, a scaffold and adaptor protein that binds both polyubiquitinated proteins destined for degradation and LC3 on autophagosomal membranes. Western blotting experiments revealed that exposure of cells or purified p62 to verteporfin causes the formation of covalently crosslinked p62 oligomers by a mechanism involving low-level singlet oxygen production. Rose bengal, a singlet oxygen producer structurally unrelated to verteporfin, also produced crosslinked p62 oligomers and inhibited autophagosome formation. Co-immunoprecipitation experiments demonstrated that crosslinked p62 oligomers retain their ability to bind to LC3 but show defective binding to polyubiquitinated proteins. Mutations in the p62 PB1 domain that abolish self-oligomerization also abolished crosslinked oligomer formation. Interestingly, small amounts of crosslinked p62 oligomers were detected in untreated cells, and other groups noted the accumulation of p62 forms with reduced SDS-PAGE mobility in cellular and animal models of oxidative stress and aging. These data indicate that p62 is particularly susceptible to oxidative crosslinking and lead us to propose a model whereby oxidized crosslinked p62 oligomers generated rapidly by drugs like verteporfin or over time during the aging process interfere with autophagy.

## Introduction

Autophagy is a cellular catabolic process in which macromolecules and organelles are sequestered into double-membraned vesicles called autophagosomes fuse with lysosomes for degradation and recycling. Autophagy maintains homeostasis by turning over cytoplasmic constituents and responding to the metabolic state of the cell. Extracellular and intracellular stresses including starvation, hypoxia, pathogen invasion, and accumulation of damaged proteins and organelles stimulate autophagy as a cytoprotective response. Genetic deletion and knockdown experiments have implicated autophagy in the origin and progression of a number of pathophysiological states including cancer, neurodegeneration, myopathies, and infectious diseases [1], [2]. The emerging role of autophagy in health and disease has stimulated interest in identifying small-molecule stimulators and inhibitors of autophagy [3], [4]. A phenotypic cell-based screen for autophagy inhibitors revealed verteporfin, an approved drug, as an inhibitor of autophagosome formation [5]. Clinically, verteporfin is used in photodynamic therapy to treat macular degeneration [6], but has been shown to inhibit autophagy in the absence of light activation both in cells and *in vivo* [5], [7]. The mechanism of action of verteporfin is unknown but it appears to inhibit autophagosome formation at a step following the recruitment of lipidated LC3 to membranes but preceding cargo sequestration into double-membraned vesicles [5].

p62 is a multifunctional scaffold protein with diverse cellular functions arising from its ability to interact with a number of proteins involved in various signaling and regulatory pathways including autophagy. As an adaptor protein for selective autophagy, p62 binds ubiquitinated cargo through its C-terminal UBA domain [8] and it binds lipidated LC3, a protein stably associated with the autophagosome membrane, via its LC3-interacting region [9], [10]. These interactions facilitate the sequestration of ubiquitinated targets into autophagosomes for lysosomal degradation. p62 has an N-terminal Phox/Bem 1p (PB1) domain that governs its oligomerization [11]–[13]. Binding between PB1 domains is mediated by electrostatic interactions between the basic surface of one and the acidic surface of another. Most proteins in the PB1 family contain either a basic motif or an acidic motif; but the PB1 domain in p62 has one of each on opposite faces. This feature allows p62 to self-oligomerize and contributes to its versatile cellular function [14]–[16].

In this study, we characterize p62 as a target of verteporfin. We report that verteporfin inhibits p62 function by a mechanism involving the formation of high-MW forms of p62 that resist denaturation. *In vitro* experiments show that verteporfin acts directly on p62, generating covalently crosslinked oligomers via low level singlet oxygen. Moreover, we determined that high-MW p62 formation is dependent on oligomerization through the PB1 domain and that it does not affect association with EGFP-LC3 but impairs binding to polyubiquitinated cargo. Based on this data, we present a model for verteporfin-mediated inhibition of autophagosome formation via abnormal p62 crosslinking.

## Experimental procedures

### Reagents

Reagents were purchased as follows: cell culture reagents from Sigma-Aldrich unless stated otherwise; general laboratory chemicals from Sigma-Aldrich, Fisher Scientific, and BDH Inc.; rose bengal from Sigma-Aldrich (330000); bafilomycin A1 (B1080) from LC Laboratories; verteporfin from Prestwick Chemical or from the United States Pharmacopeial Convention, Inc.; peroxynitrite (81565) and diethylamine-NONOate (DEA/NONOate 82100) from Cayman Chemicals; H_2_O_2_ (HX0635–2) from EM Science; NaOCl (SS290) from Fisher Scientific.

### Cell culture procedures

MCF-7 cells stably transfected with pEGFP-LC3 [3] and BxPC-3 cells [7] were maintained in RPMI 1640 supplemented with 1 mM Hepes, 10% (v/v) fetal bovine serum (FBS), 100 units/ml penicillin and 100 µg/mL streptomycin (Gibco). EGFP-LC3-expressing cells were supplemented with 400 µg/ml G418. All MEF cell lines [17] were maintained in DMEM supplemented with 10% FBS, 1 mM sodium pyruvate (Gibco 11360-070), and 1X MEM non-essential amino acids (Gibco 11140-050). Tet-On cell lines were also supplemented with 10 µg/ml blasticidin. GFP-p62 wt and GFP-p62 K7A/D69A expression was induced with 50 µg/mL doxycycline for 1–4 h or 24 h, respectively.

### Automated assay for monitoring autophagosome accumulation

Punctate EGFP-LC3 was monitored and quantified in MCF-7 EGFP-LC3 cells as described previously [3], [5].

### SDS-PAGE and immunoblotting

Cells were harvested in 20 mM Tris-HCl, pH 7.5, 150 mM NaCl, 1% (v/v) Triton X-100, 1 mM EDTA, 1 mM EGTA, 2.5 mM sodium pyrophosphate, 1 mM β-glycerophosphate, 1 mM sodium orthovanadate, and 1x complete protease inhibitor cocktail (Roche 1169748001). Lysates were centrifuged at 18,000×g at 4°C, and supernatants were collected and quantified by Bradford assay (Bio-Rad). Lysates were normalized for protein content in SDS-PAGE sample buffer and analyzed by immunoblotting as in [3], [5]. For immunodetection of polyubiquitin, nitrocellulose membranes were boiled for 10 min following protein transfer and prior to blocking. Primary antibodies used were mouse α-GFP (1∶7000, Roche 11814460001), mouse α-SQSTM1/p62 (1∶250, Santa Cruz, sc-28359, for human cell lysates), mouse α-p62 (1∶15000, Abnova, H00008878, for MEF cell lysates), mouse α-polyubiquitin (1∶1000, Santa Cruz Biotechnology, Inc sc-53509), and rabbit α-β-tubulin (1∶20000, Santa Cruz, sc-9104). The secondary antibodies used were HRP-conjugated goat α-mouse IgG, Light Chain Specific (1∶10000, Jackson ImmunoResearch 115-035-174), HRP-conjugated goat α-mouse IgG (1∶10000, ThermoScientific 31430), and HRP-conjugated goat α-rabbit IgG (1∶10000, KPL 074-1506).

For immunodetection of oxidized proteins, the Oxyblot Immunodetection Kit (Millipore s7150) was used according to the manufacturer’s instructions. Briefly, proteins samples were derivatized with DNPH prior to western blotting using primary α-DNP and secondary antibodies diluted in 1% BSA in PBS-T at 1∶150 and 1∶300, respectively.

### Purification of recombinant p62

BL21 bacteria expressing GST-p62 from a pGex-4T-1 expression plasmid with p62 inserted at the NotI and SalI sites were a kind gift from Dr. Thibault Mayor. GST-p62 was expressed by induction with 0.1 mM IPTG at 37°C for 3 h. The cells were lysed by sonication in PBS+1% Tx-100, pH 7.4. Insoluble proteins were removed by centrifugation at 13,000 rpm for 15 min at 4°C. The cell extract was incubated with glutathione sepharose 4B (GE 17-0756-01) rotating end-over-end for 4 h at 4°C followed by gravity flow column purification. The column was washed twice with PBS+1% Tx-100 and twice with PBS. GST-p62 was eluted with freshly prepared 50 mM Tri-HCl, 10 mM glutathione, pH 8.0 followed by buffer exchanger with 50 mM Tris-HCl, 50 mM NaCl, 10 mM glutathione, pH 9.0 using 30,000 NMWL Amicon Ultra-15 centrifugal filter units.

### *In vitro* recombinant p62 assays

Purified recombinant His-p62 (NBP-44490) was from Novus Biologicals. The buffer used for all recombinant p62 reactions was 50 mM Tris-HCl, 150 mM NaCl, pH 7.5. For immunoblot analysis, 50 ng GST-p62 or 26.3 ng His-p62 was treated as described. Reactions were stopped by the addition of 4X SDS-PAGE sample buffer. ROS reactions were carried out as follows: GST-p62 was exposed to the indicated molar ratios of NaOCl or H_2_O_2_ for 1 h at 37°C and the reactions were stopped with 100 mM methionine [18]; GST-p62 was exposed to increasing concentrations of peroxynitrite for 5 min at room temperature as previously described [19]; GST-p62 was exposed to increasing concentrations of DEA/NONOate for 20 min at room temperature as previously described [20]. Peroxynitrite and DEA/NONOate dilutions were prepared fresh in 0.1 M NaOH and 0.01 M NaOH, respectively, and the appropriate NaOH controls were carried out.

### Immunoprecipitation

Cell lysates were incubated with mouse α-SQSTM1/p62 or mouse α-GFP for 4 h at 4°C, followed by overnight incubation with Protein-G-agarose beads (Roche Molecular Biochemicals 1124323301) at 4°C. Immunoprecipitates were washed three times with lysis buffer at 4°C. Samples were eluted by boiling beads in 2X SDS-PAGE sample buffer containing 50 mM DTT for 10 min, proteins were detected by western blotting.

### Immunofluorescence microscopy

MCF-7 EGFP-LC3 cells were seeded on 7X detergent-treated glass coverslips in 12-well plates at 300,000 cells/well in normal culture medium and were treated the next day as indicated. Cells were then washed once with PBS and fixed and permeabilized in 100% methanol at −20°C for 6 min. After multiple PBS washes to remove methanol, cells were blocked in 1% bovine serum albumin (BSA) in PBS for 10 min. The cells were incubated with mouse α-p62 antibody in 3% BSA in PBS for 45 min, washed twice with PBS, incubated in goat α-mouse AlexaFluor 568 secondary antibody (1∶1000, Molecular Probes) in 3% BSA in PBS for 45 min, and then washed twice with PBS. DNA was stained with Hoechst 33342 at 500 ng/ml for 5 min. Coverslips were mounted on glass slides using CelVol and viewed using the 60X objective of an Olympus Fluoview FV1000 laser scanning microscope equipped with Olympus-selected Hamamatsu photomultiplier tubes. Images were analyzed using Olympus FV10-ASW1.7 software.

## Results

### Induction of high-molecular weight p62 by the autophagy inhibitor verteporfin

**Figure 1:**
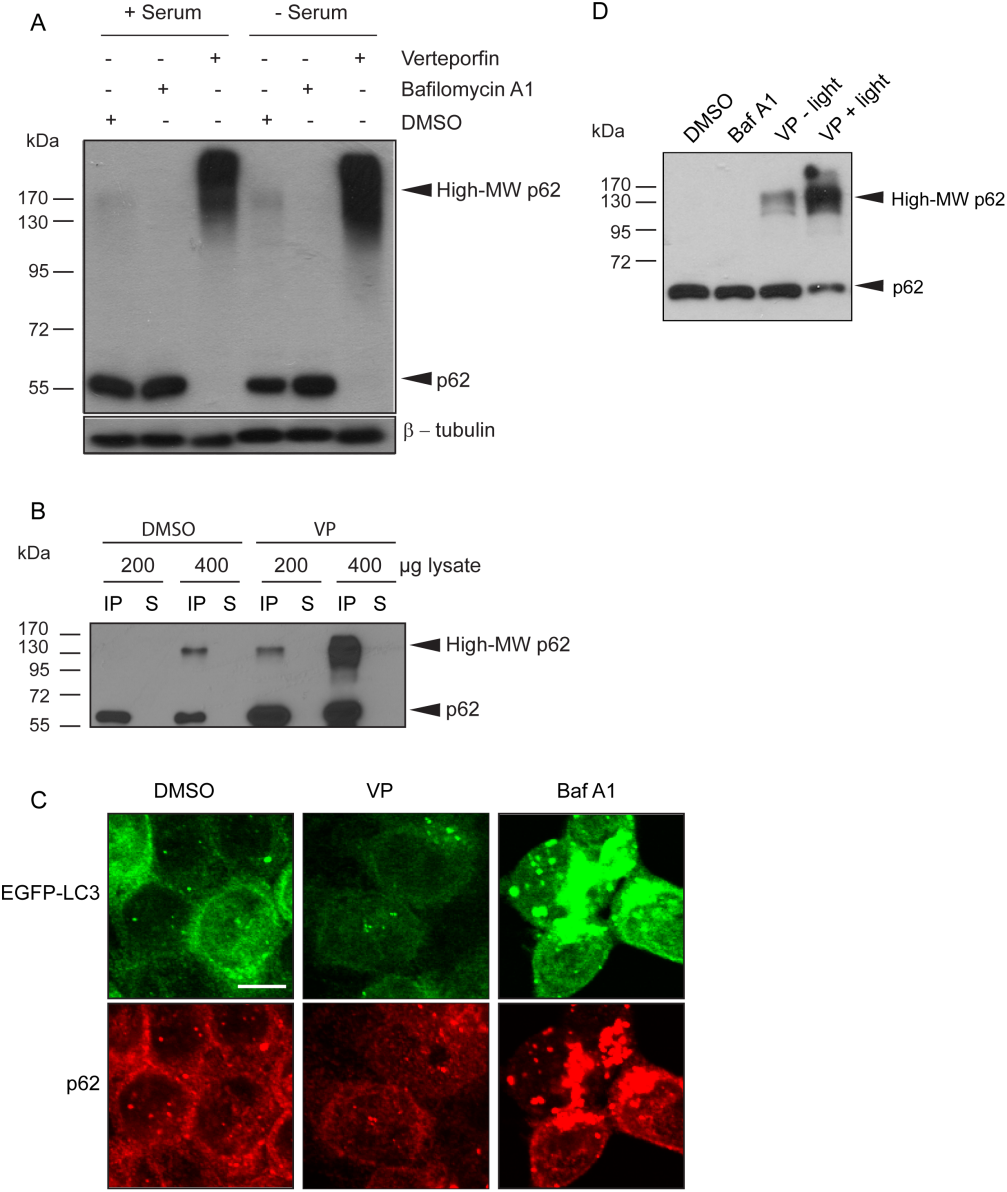
Effect of verteporfin on p62 in cells. (A) MCF-7 EGFP-LC3 cells were exposed to 0.1% DMSO, 100 nM bafilomycin A1, or 10 µM verteporfin for 8 h in the presence or absence of serum and cell lysates were immunoblotted for p62. β-tubulin was monitored as a loading control. (B) MCF-7 EGFP-LC3 cells were exposed to 0.1% DMSO or 10 µM verteporfin for 4 h. Indicated amounts of each lysate were immunoprecipitated with anti-p62 antibody and analyzed by western blot. (C) MCF-7 EGFP-LC3 cells were exposed to 0.1% DMSO, 10 µM verteporfin, or 100 nM bafilomycin A1 for 4 h in complete medium. The cells were fixed and stained with p62 antibody, and images were acquired by confocal microscopy. Scale bar, 10 µm. (D) MCF-7 EGFP-LC3 cells were exposed to 0.1% DMSO, 100 nM bafilomycin A1, or 10 µM verteporfin for 4 h in complete medium. Cell lysates were collected, quantified, and normalized in the presence or absence of overhead laboratory light as indicated. 0.5 µg of lysate was used to examine p62 levels by western blotting. All images presented are representative of at least 3 independent experiments.

p62 is a multifunctional protein commonly used to monitor autophagic flux [21]. As an adapter protein, p62 tethers polyubiquitinated proteins destined for degradation to the membrane of nascent autophagosomes via LC3 and it is degraded along with its cargo [22], [23]. In a recent study on the therapeutic effect of verteporfin in a pancreatic cancer xenograft model, we observed that verteporfin caused the appearance of an altered form of p62 that showed reduced electrophoretic mobility in denaturing SDS-PAGE conditions, which we denoted as high-MW p62 [7]. To characterize this unusual form of p62 and to investigate the mechanism of action of verteporfin, we first compared its effect on p62 levels to those of bafilomycin A1, a v-ATPase inhibitor that prevents autophagosomal degradation. When MCF-7 cells, which display low basal autophagy in complete cell culture medium containing serum [5], were exposed to 100 nM bafilomycin A1 for 8 h, there was no visible increase in cellular p62 levels compared to untreated cells (Fig. 1A), confirming low basal autophagy. Exposing cells to serum-free medium to stimulate autophagic flux caused a noticeable decrease in p62 levels that was prevented by co-incubation with 100 nM bafilomycin A1 (Fig. 1A), demonstrating degradation of p62 by autophagy under these conditions.

As an inhibitor of autophagy, verteporfin was similarly expected to prevent p62 degradation under autophagy-stimulating conditions. However, when MCF-7 cells were exposed to verteporfin in complete medium or in serum-free medium, we observed that p62 did not accumulate at 60 kDa, but as a smear starting at ∼120 kDa and surpassing the highest MW band of the protein ladder at 170 kDa (Fig. 1A, *High-MW p62*). Bafilomycin A1 did not induce high-MW p62, showing that high-MW p62 production was not merely a consequence of inhibiting autophagy (Fig. 1A).

Both 60-kDa and high-MW p62 were effectively immunoprecipitated by a p62 antibody from cells treated for 4 h with verteporfin (Fig. 1B). Interestingly, when p62 was immunoprecipitated from vehicle-treated control cells, a small amount of high-MW p62 was detected when sufficient material was loaded (Fig. 1B, *third lane*) implying the presence of very low amounts of this form under physiological conditions. Immunoprecipitates from verteporfin-treated cells contained larger amounts of both 60-kDa and high-MW p62 than DMSO-treated controls, demonstrating that verteporfin both prevents p62 degradation and causes its modification to the high-MW form (Fig. 1B).

Several studies have shown that p62 is a common component of cellular protein aggregates including Lewy bodies, huntingtin aggregates, Mallory bodies, and hyaline bodies [24]–[26], and many of these p62 aggregates have been identified as autophagy substrates [10], [23], [27], [28]. Since it was previously demonstrated that ubiquitin- and p62-positive inclusion bodies accumulate in autophagy-deficient cells [29]–[31], it was important to determine whether high-MW p62 accumulates in cells as aggregates as a consequence of verteporfin-mediated autophagy inhibition. p62 localization was analyzed by immunofluorescence microscopy in MCF-7 cells stably expressing EGFP-LC3. Exposure to bafilomycin A1 caused a significant increase in punctate p62 immunofluorescence (Fig. 1C) that largely colocalized with punctate EGFP-LC3, indicative of undigested autophagosomes containing p62-tethered cargo, as expected. By contrast, 4 h treatment with 10 µM verteporfin did not increase punctate p62 immunofluorescence, implying that verteporfin does not induce p62 aggregates (Fig. 1C). As expected, verteporfin did not affect EGFP-LC3 localization since the cells were treated in complete medium where basal autophagy is low (Fig. 1C). Since verteporfin prevents autophagosome formation, the vehicle for p62 sequestration and delivery for lysosomal degradation is no longer present in cells and its localization is largely unchanged after verteporfin treatment (Fig. 1C).

It is important to note that initial investigations into the effects of verteporfin on p62 showed variable proportions of high-MW p62 to monomeric p62. High-MW p62 was always present after verteporfin treatment but in some experiments no 60-kDa p62 was detected in verteporfin-treated cells (Fig. 1A) while in other experiments both 60-kDa and high-MW forms of p62 were observed (Fig. 1B,D). The explanation for these differences was traced to differences in light exposure during sample handling. Verteporfin is a photoactivatable molecule and exposure to light was always stringently avoided during cell treatment with verteporfin. However, light exposure was initially not as tightly controlled during cell lysis and subsequent experimental steps. To address any effects of light exposure, MCF-7 EGFP-LC3 cells were treated with 10 µM verteporfin for 4 h in the dark followed by lysis without or with overhead fluorescent ambient laboratory lighting. Both verteporfin-treated samples contained high-MW p62 that was not present in DMSO- or bafilomycin A1- treated cells. However, the verteporfin-treated cell lysate exposed to ambient light showed a stronger and broader high-MW p62 band as well as noticeably less of the 60-kDa form compared to the verteporfin sample not exposed to light (Fig. 1D). These results suggest that the effect of verteporfin on p62 occurs in the absence of illumination, and it can be amplified by exposure to ambient light after cell lysis. Accordingly, exposure to light was carefully avoided in all subsequent experiments unless otherwise noted.

### Production of high-molecular weight p62 *in* *vitro*

**Figure 2:**
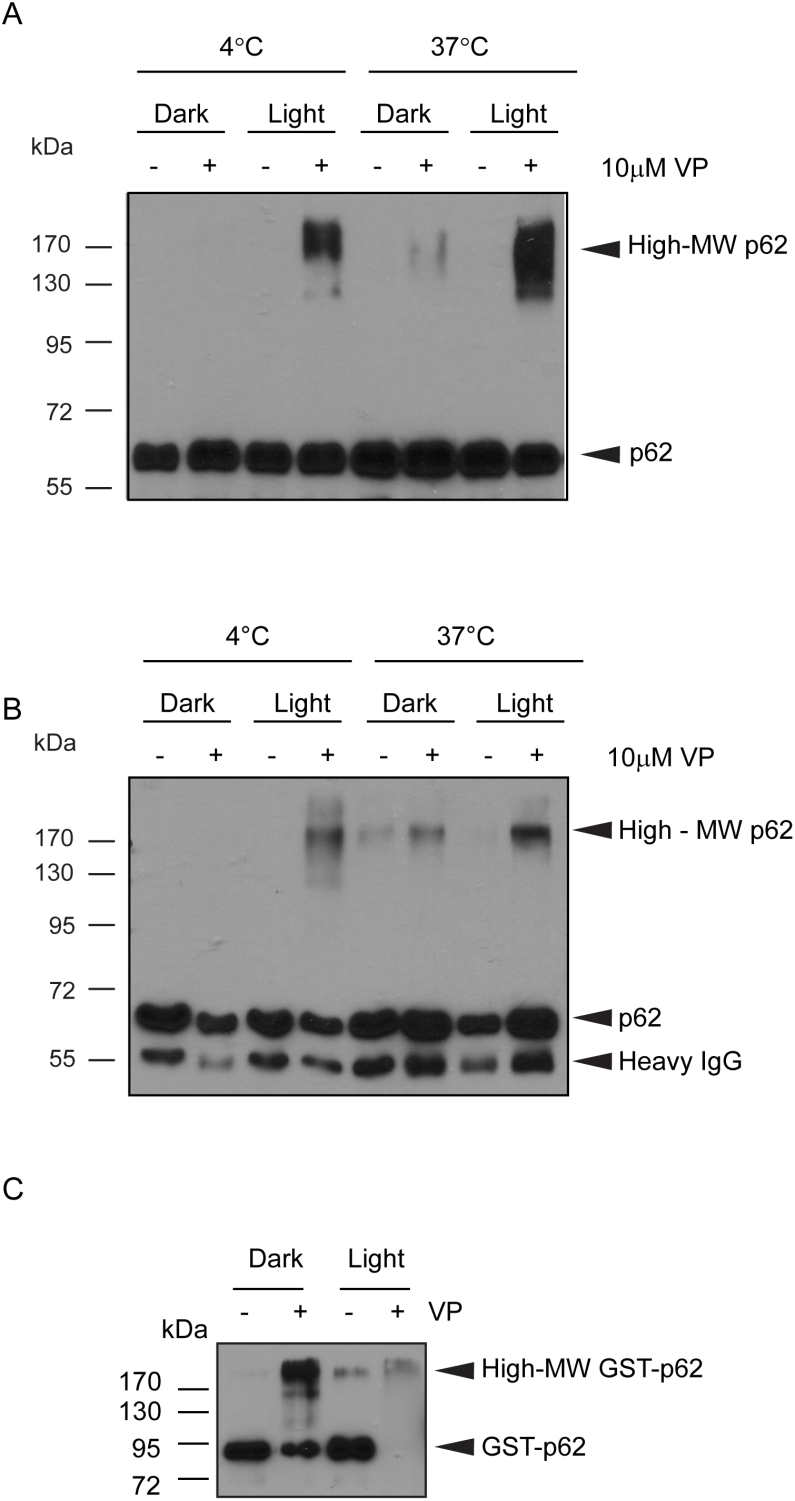
Effect of verteporfin on p62 *in vitro*. (A) Equal amounts of untreated BxPC-3 cell lysate were exposed to 10 µM verteporfin in the presence or absence of light at 4°C or 37°C for 30 min. (B) p62 was immunoprecipitated from untreated BxPC-3 cells. The immunoprecipitated material was then treated for 30 min in lysis buffer with 10 µM verteporfin in the presence or absence of light at 4°C or 37°C. (C) 100 ng purified GST-p62 was exposed to 10 µM verteporfin for 1 h at 37°C in the absence or presence of light. The above reactions were all immunoblotted for p62. All images presented are representative of at least 3 independent experiments.

To determine whether the formation of high-MW p62 was a direct effect of verteporfin on p62 or resulted from a cellular response to treatment, lysates prepared from untreated BxPC-3 cells were incubated without or with 10 µM verteporfin for 30 min at 4°C or 37°C in the absence (dark) or in the presence of overhead fluorescent ambient light (light) in the laboratory. Immunoblotting for p62 showed that in the absence of verteporfin, only the 60-kDa form was present after incubation at 4°C or 37°C in light or dark conditions. In the presence of light at 4°C, verteporfin generated a range of high-MW p62 forms appearing above the 130 kDa protein marker and spanning beyond 170 kDa (Fig. 2A). Exposure to verteporfin at 37°C in the absence of light induced high-MW p62 spanning the same range, but the smear was much less intense. Exposure of cell lysate to verteporfin at 37°C in the presence of light produced the most intense and broadest range of high-MW p62 forms starting from ∼110 kDa and spanning beyond 170 kDa (Fig. 2A). Therefore, verteporfin is capable of generating high-MW p62 in cell lysates *in vitro* in the presence of thermal or light energy. The fact that verteporfin generated high-MW p62 in the presence of light at 4°C implies that enzymatic mechanisms do not confer this modification. Moreover, the observation that light or heat was required to induce high-MW p62 by verteporfin suggests that high-MW p62 is the product of a chemical modification rather than a conformational change or an oligomerization product.

To further investigate this effect, p62 was immunoprecipitated from untreated cells, washed extensively and then treated with 10 µM verteporfin in the presence or absence of light at 4°C or 37°C. Untreated p62 immunoprecipitates incubated at 4°C in the dark or light contained only 60-kDa p62, but exposure to verteporfin and light at 4°C generated high-MW p62 (Fig. 2B). Interestingly, incubating untreated immunoprecipitate at 37°C in the dark or light generated a small amount of high-MW p62, detected as a faint smear near 170 kDa. However, exposing p62 immunoprecipitates to verteporfin under the same conditions generated noticeably more high-MW p62 forms than observed in the respective untreated samples (Fig. 2B). Immunoprecipitated p62 was also modified *in vitro* by verteporfin in the presence of stringent detergents (data not shown), implying that verteporfin affects p62 directly or through a very strongly associated protein(s).

To determine whether verteporfin acts directly on p62, GST-p62 produced in *E. coli* was purified and incubated in the presence or absence of 10 µM verteporfin for 1 h at 37°C in the dark or light. In the absence of light, untreated GST-p62 was detected as a single 80 kDa band, but exposure to verteporfin decreased the intensity of the 80 kDa band and generated an intense high-MW band, detected above the largest MW marker at 170 kDa (Fig. 2C). Similar to the previous experiment, a small amount of high-MW p62 was observed in the untreated GST-p62 sample after exposure to light and heat, but the majority of p62 was detected at 80 kDa. These observations demonstrate that verteporfin treatment in the absence of light at 37°C directly affects p62 and confers a modification or crosslink that increases the molecular weight significantly. When GST-p62 was exposed to verteporfin in the presence of both light and heat, its signal almost completely disappeared from the blot (Fig. 2C). We speculate that the combination of verteporfin, heat, and light, each of which alone can modify p62, either caused its degradation or prevented it from entering the gel.

In summary, both heat and light cause verteporfin to form high-MW p62 and the banding pattern is reproducible regardless of the energy source. Since the extensively washed immunoprecipitates and the purified GST-p62 was exposed to verteporfin in buffered salts only, verteporfin must act directly on p62, without any need for other proteins, enzymatic activity, or energy from ATP.

### Verteporfin-mediated high-MW p62 is due to protein oxidation by singlet oxygen

Verteporfin is known to produce large amounts of singlet oxygen (^1^O_2_) upon laser light irradiation at 690 nm [32]–[34]. Singlet oxygen (^1^O_2_) is a type of reactive oxygen species (ROS) generated by an input of energy, classically photodynamic activation, that results in a rearrangement of electrons in the oxygen atom [35]. While singlet oxygen has the ability to oxidize a number of biological molecules [36], [37], proteins are the primary target due to their cellular abundance and because singlet oxygen rapidly reacts with the residues tryptophan, histidine, tyrosine, cysteine, and methionine at physiological pH [37]–[39]. These oxidation products are then capable of crosslinking with other residues to produce interprotein crosslinks, with His-His, His-Lys, His-Arg, His-Cys, and Tyr-Tyr interactions the most commonly reported [37], [40]–[43], thus generating high-molecular mass products [44], [45]. In most studies, protein crosslinking by singlet oxygen producers has been observed only upon intense light irradiation [41], [46], but Bae *et al.* [47] showed that in the presence of some drugs, protein crosslinking can occur after brief exposure to laboratory light, as we observed with p62 and verteporfin. Therefore, we hypothesize that thermal energy is sufficient to activate verteporfin to generate a low level of singlet oxygen, and that this is increased by even low levels of light.

**Figure 3:**
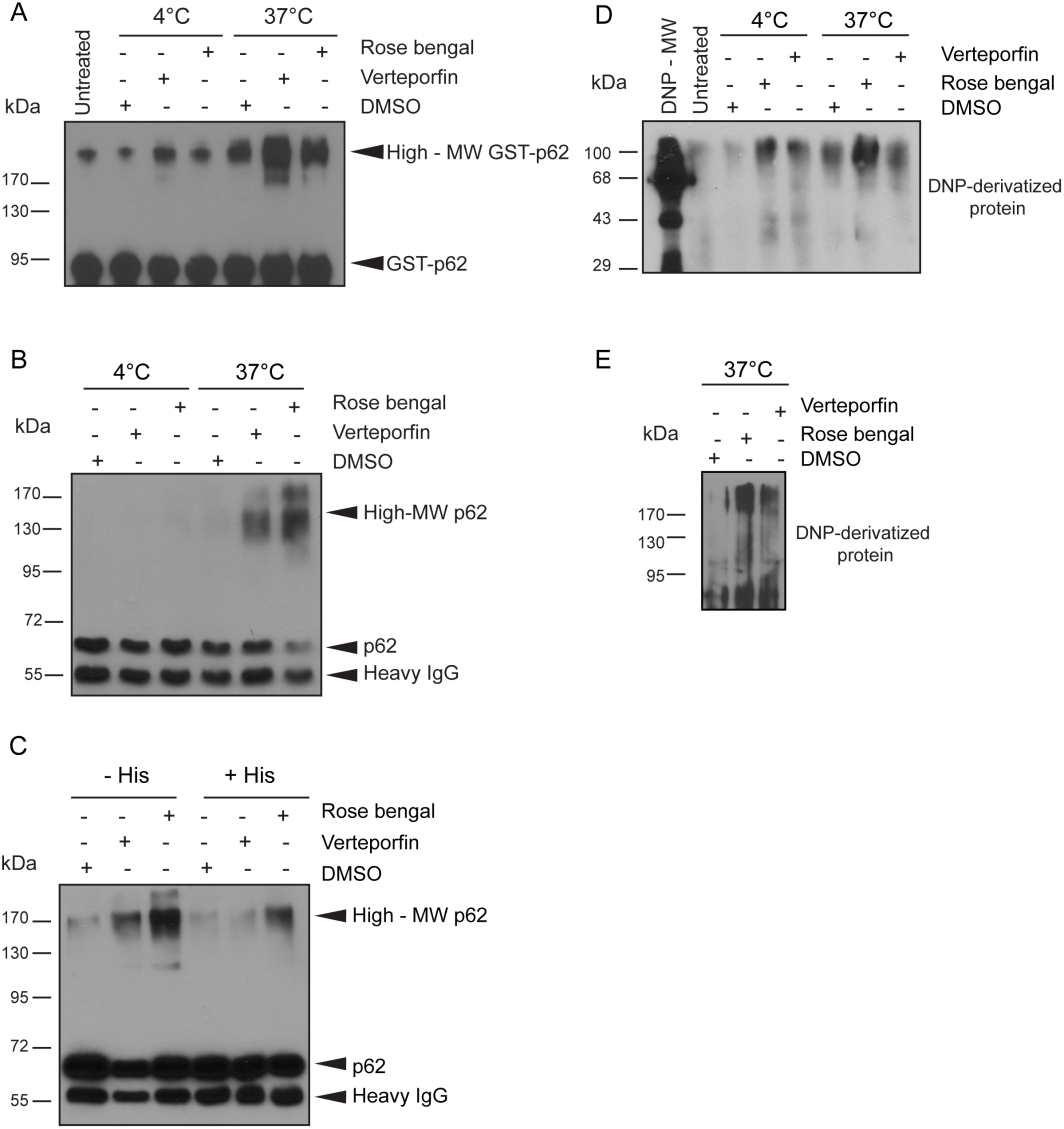
High-molecular weight p62 induced by singlet oxygen production. (A) 50 ng GST-p62 was incubated with 0.05% DMSO, 5 µM verteporfin, or 5 µM rose bengal for 1 h at 4°C or 37°C in the dark. (B) Immunoprecipitated p62 from untreated BxPC-3 cells was exposed to 0.1% DMSO, 10 µM verteporfin, or 10 µM rose bengal for 30 min in the dark at 4°C or 37°C or to (C) 0.1% DMSO, 10 µM verteporfin, or 10 µM rose bengal in the dark at 37°C with or without 20 mM histidine. Reactions were immunoblotted for p62. (D) 26 ng purified His-p62 was incubated with 0.05% DMSO, 5 µM verteporfin, or 5 µM rose bengal for 1 h at 4°C or 37°C in the dark or (E) 50 ng purified GST-p62 was incubated with 0.1% DMSO, 10 µM verteporfin, or 10 µM rose bengal for 30 min at 37°C in the dark. Samples were derivatized with DNPH, subjected to SDS-PAGE, and immunoblotted with anti-DNP antibody solution according to the Oxyblot kit manufacturer’s instructions. All images presented are representative of at least 3 independent experiments.

To test this hypothesis, we examined the effects of rose bengal, a widely used chemical dye that produces singlet oxygen and is structurally unrelated to verteporfin [41], [43], [48]. GST-p62 was exposed to 5 µM rose bengal or 5 µM verteporfin for 1 h at 4°C or 37°C in the dark (Fig. 3A). A small amount of high-MW GST-p62 was observed in the DMSO control after exposure to heat, again demonstrating that heat alone can generate some high-MW p62 *in vitro*. Exposure of recombinant p62 to rose bengal at both 4°C and 37°C induced high-MW p62 forms similar to those generated by verteporfin under the same conditions, and combining rose bengal treatment with heat strongly stimulated the formation of high-MW GST-p62 (Fig. 3A). The fact that significant amounts of high-MW GST-p62 forms were generated in reactions containing only recombinant protein, buffered salt, a photosensitizer, and heat indicates that singlet oxygen production causes p62 oxidation and subsequent crosslinking. The *in vitro* effect of rose bengal was also demonstrated on cellular p62. p62 was immunoprecipitated from untreated cells and then treated with 10 µM rose bengal or 10 µM verteporfin for 30 min at 4°C or 37°C in the dark. Neither had any effect at 4°C, but at 37°C immunoprecipitated p62 was modified by rose bengal to generate a range of high-MW forms as seen with verteporfin treatment (Fig. 3B).

The effects of singlet oxygen can be quenched *in vitro* using excess histidine [36], [47], [49]. The imidazole side chain of histidine has been shown to be one of the most vulnerable to modification by photooxidation via singlet oxygen [39], [50] and excess histidine added in the presence of singlet-oxygen acts as an abundantly available substrate for oxidation, thus reducing cellular protein oxidation and subsequent reactions [37]. p62 was immunoprecipitated from untreated BxPC-3 cells and then treated with 10 µM verteporfin or 10 µM rose bengal in the presence or absence of 20 mM histidine for 30 min at 37°C. High-MW p62 was detected in all treatments lacking histidine, even the DMSO-treated control although at a very low level compared with the rose bengal and verteporfin treatments (Fig. 3C). The intensity of the high-MW p62 signal was decreased in all samples containing histidine, supporting the hypothesis that high-MW p62 generated by verteporfin is mediated by low levels of singlet oxygen generation.

It has been shown that singlet oxygen-mediated protein oxidation is characterized, in part, by conversion of some amino acid residues into carbonyl derivatives [51]–[53]. Carbonyl content can be measured by derivatization with 2,4-dinitrophenylhydrazine (DNPH), which reacts with ketones and aldehydes to produce stable DNP-hydrazone amino acid products that are specifically detected with anti-DNP antibodies [53]–[55]. Using this “Oxyblot” procedure, His-tagged p62 was exposed to 5 µM rose bengal or 5 µM verteporfin for 1 h at 4°C or 37°C in the dark. An equal amount of untreated His-p62 was also analyzed (Fig. 3D, untreated). At 4°C very little signal was detected in the untreated and vehicle controls. Exposure to either rose bengal or verteporfin at 4°C showed a significant increase in carbonyl content at ∼40 kDa, the size of His-p62, and at the high MW-region ≥90 kDa, (Fig. 3D). When the same treatments were carried out at 37°C the carbonyl signal was entirely localized to the high-MW region ≥90 kDa, and exposure to rose bengal clearly increased the intensity. Under these conditions, verteporfin did not produce a detectable increase in carbonyl content; therefore, the experiment was repeated at 37°C with GST-p62. GST-p62 exposed to 10 µM rose bengal or 10 µM verteporfin for 30 min at 37°C in the dark showed a significant increase in detected carbonyl residues at both 80 kDa and>170 kDa, the respective sizes of GST-p62 and high-MW GST-p62 (Fig. 3E). The presence of oxidized high-MW His-p62 and GST-p62 after incubation at 37°C in the DMSO control confirmed previous findings that heat alone can generate high-MW p62 and demonstrated a correlation with its production and an increase in protein oxidation. Of the amino acids with which singlet oxygen has a tendency to react, histidine and tryptophan are the most likely to be converted directly into carbonyl derivatives at physiological pH [37], [39], [50], and it has been shown that the formation of interprotein crosslinks via oxidized His residues produces more carbonyl derivatives in the process [37], [38], [56]. The increased intensity of oxidized His-p62 and GST-p62 under conditions that have consistently produced high-MW p62 provides substantial evidence that verteporfin-induced high-MW p62 is a product of singlet oxygen-mediated crosslinking.

### Induction of high-MW p62 by other sources of reactive oxygen species

**Figure 4:**
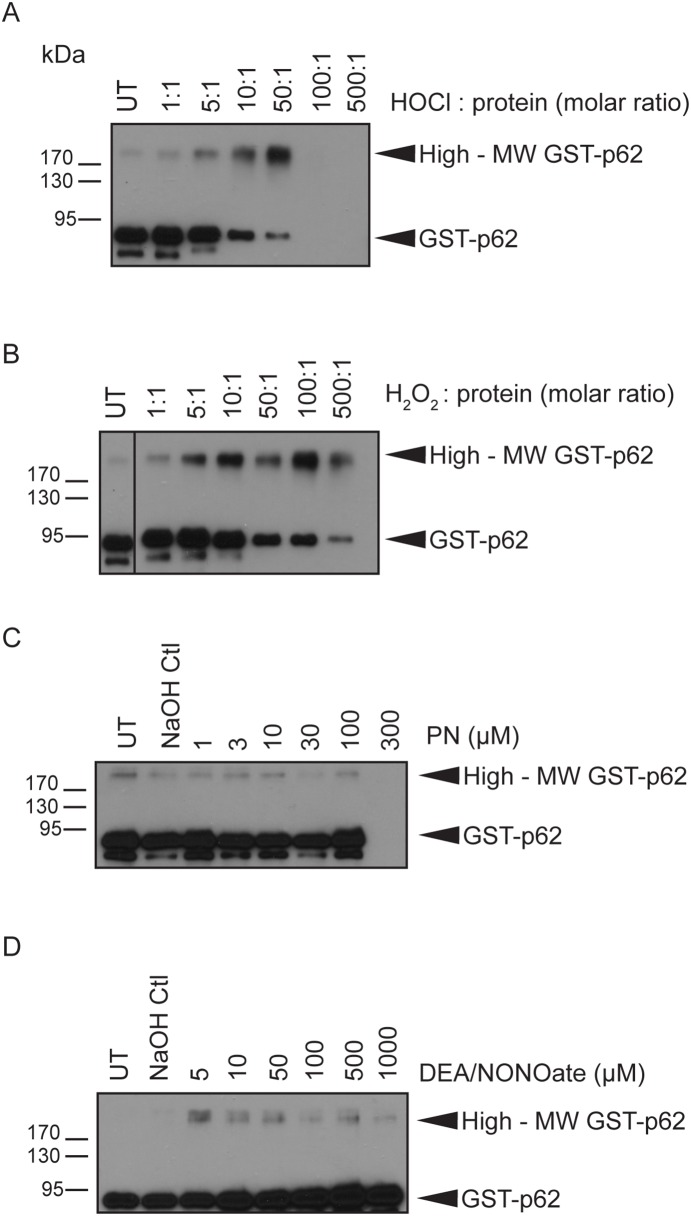
Effect of different ROS sources on p62 *in vitro*. 50 ng purified GST-p62 was exposed to different molar ratios of (A) NaOCl or (B) H_2_O_2_ for 1 h at 37°C, or to different concentrations of (C) peroxynitrite for 5 min at room temperature or (D) DEA/NONOate for 20 min at room temperature. All reactions were immunoblotted for p62. All images presented are representative of at least 3 independent experiments.

To examine whether other reactive oxygen species generate high-MW p62 *in vitro*, GST-p62 was exposed to a panel of oxidants: hydrogen peroxide, hypochlorous acid, peroxynitrite, and nitric oxide. GST-p62 was exposed to HOCl or to H_2_O_2_ at increasing molar ratios (oxidant:protein) for 1 h at 37°C. A small amount of high-MW GST-p62 was observed in the untreated sample (Fig. 4A–B). HOCl generated increasing amounts of high-MW GST-p62 from molar ratios 5∶1 to 50∶1 with a corresponding decrease in 80-kDa GST-p62. No protein was observed at higher HOCl concentrations, suggesting that the protein degraded in these conditions (Fig. 4A). Similarly H_2_O_2_ produced increasing amounts of high-MW GST-p62 from molar ratios 5∶1 to 100∶1, but the total protein signal decreased at 500∶1 suggesting that protein degradation occurred at this molar ratio (Fig. 4B). Peroxynitrite is a potent oxidant and nitrating agent that has a very short half-life (<1 sec) [57]; therefore, GST-p62 was exposed to increasing concentrations of peroxynitrite for only 5 min at room temperature. A small amount of high-MW GST-p62 was observed in both the untreated and NaOH control, and this did not increase in the presence of peroxynitrite (Fig. 4C). No protein was detected in the presence of 300 µM peroxynitrite, again indicating that extremely large amounts of oxidants cause protein degradation (Fig. 4C). Finally, GST-p62 was exposed to different concentrations of DEA/NONOate, a nitric oxide donor [20], for 20 min at room temperature. In this experiment, no high-MW GST-p62 was observed in the controls, but exposure to 5 µM DEA/NONOate generated a faint high-MW GST-p62 band. However, unlike HOCl and H_2_O_2_,DEA/NONOate did not produce a concentration-dependent increase in high-MW GST-62 (Fig. 4D). Taken together, these experiments show that p62 is highly susceptible to oxidation *in vitro* leading to the formation of high-MW p62 by singlet oxygen, HOCl, and H_2_O_2_, and it is much less reactive towards nitrating oxidants, peroxynitrite and nitric oxide.

### Rose bengal induces high-MW p62 in cells and inhibits starvation-induced autophagosome accumulation

**Figure 5:**
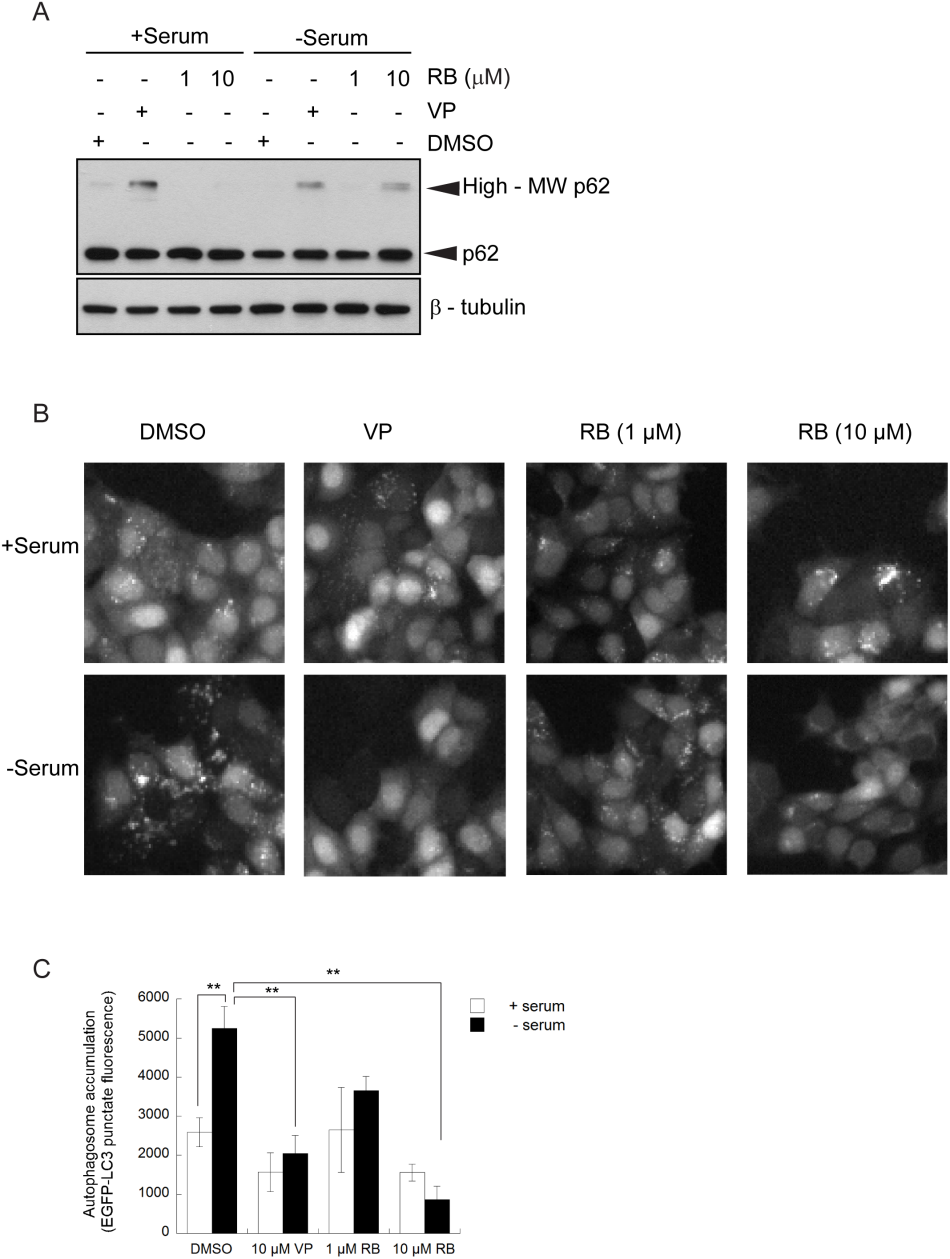
Effect of rose bengal on autophagosome accumulation and p62 in cells. MCF-7 EGFP-LC3 cells were exposed for 4 h to 0.1% DMSO, 10 µM verteporfin (VP), or different concentrations of rose bengal (RB) in the presence or absence of serum. (A) Cell lysates were immunoblotted for p62 and β-tubulin. This image is representative of 2 independent experiments. (B) Cells were fixed and stained with Hoechst 33342 and punctate EGFP-LC3 fluorescence was visualized and (C) quantified using a Cellomics^VTI^ automated fluorescence microscope (**p<0.01, Student’s t-test) (mean±S.D. (error bars), n = 3).

To determine whether rose bengal can also induce high-MW p62 in cells, MCF-7 EGFP-LC3 cells were exposed to 1 or 10 µM rose bengal or to 10 µM verteporfin in the presence or absence of serum for 4 h, and p62 was monitored. In the presence of serum, a small amount of high-MW p62 was detected in the DMSO-treated control and verteporfin treatment significantly increased high-MW p62. Only 60-kDa p62 was detected in cells treated with rose bengal in complete medium (Fig. 5A). In the absence of serum and as a consequence of starvation-induced autophagy, less 60-kDa p62 was present after DMSO treatment compared to complete medium (Fig. 5A). Exposure to 10 µM verteporfin or to 10 µM rose bengal induced accumulation of both 60-kDa p62 and high-MW p62 compared to the serum-starved DMSO control. The absence of high-MW p62 in cells treated with rose bengal in complete medium is probably a consequence of its high binding to serum albumin, with 90% of the drug bound when used at 5 µM [58], [59], which would have prevented it from entering cells.

The same conditions were used to examine starvation-induced autophagosome accumulation in the presence of rose bengal. Serum starvation caused a significant increase in punctate EGFP-LC3 fluorescence compared to cells treated with DMSO in complete cell culture medium, demonstrating autophagosome accumulation in response to starvation (Fig. 5B–C). Treatment with rose bengal in serum-free medium considerably reduced starvation-induced punctate EGFP-LC3 fluorescence in a concentration-dependent fashion. Exposure of cells to either 10 µM rose bengal or 10 µM verteporfin in serum-free medium completely inhibited the accumulation of punctate EGFP-LC3 and increased diffuse cytoplasmic fluorescence (Fig. 5B–C). The fact that two chemically distinct small molecules known to generate singlet oxygen show similar autophagy modulation and p62 modification in the absence of photoactivation strongly supports a common mechanism of action.

### High-MW p62 shows reduced ability to bind to ubiquitinated proteins but not to LC3

The PB1 domain in p62 governs its self-oligomerization and interaction with a number of binding partners [11]–[13]. To provide mechanistic insight into how verteporfin-induced singlet oxygen inhibits autophagosome formation, we examined whether exposure to verteporfin affects the ability of p62 to interact with LC3 and ubiquitinated proteins.

**Figure 6:**
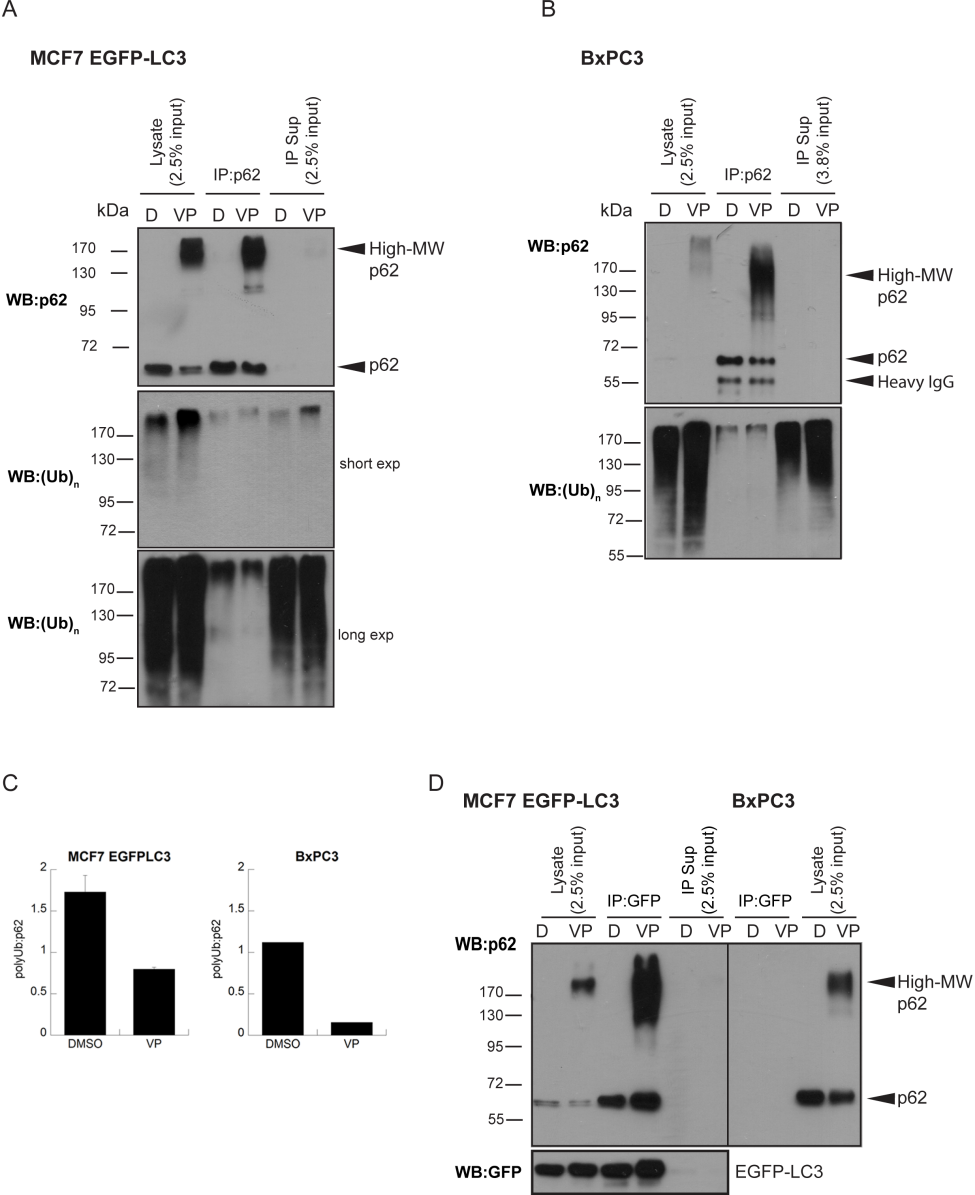
Effect of verteporfin-induced high-MW p62 on its association with polyubiquitinated proteins and LC3. (A) MCF-7 EGFP-LC3 or (B) BxPC-3 cells were exposed for 4 h to 0.1% DMSO or 10 µM verteporfin in complete medium. p62 was immunoprecipitated and the bound polyubiquitinated proteins were detected using an anti-(Ub)_n_ antibody. Immunoprecipitation was confirmed by western blot for p62. (C) Densitometry analysis was performed on the images presented in A and B using Quantity One software. (D) Using the same lysates prepared in (A) and (B), EGFP-LC3 was immunoprecipitated and bound p62 was detected in the IP fraction using an anti-GFP antibody. Immunoprecipitation was confirmed by western blot for GFP. Images presented for MCF-7 EGFP-LC3 cells are representative of at least 3 independent experiments. Densitometry was done using images from 2 of those experiments (mean ± S.D., n = 2) where the image quality was suitable for quantification. Images presented for BxPC-3 cells are representative of 2 independent experiments and densitometry was done using images from 1 experiment where the image quality was suitable for quantification.

p62 was immunoprecipitated from cells treated or not with verteporfin and immunoblotted for known interacting proteins. To investigate whether verteporfin treatment affects interaction of proteins with 60-kDa and high-MW p62, whose levels are themselves affected by drug treatment, complete immunoprecipitation of all p62 forms was ensured. Treatments were carried out in both MCF-7 EGFP-LC3 and BxPC-3 cells. In both cell lines, verteporfin treatment caused the appearance of high-MW p62 that was completely immunoprecipitated along with 60-kDa p62 from the lysates (Fig. 6A–B). p62 western blot analysis showed that nearly equal amounts of 60-kDa p62 were immunoprecipitated from DMSO- and verteporfin-treated cells while high-MW p62 was only detected in immunoprecipitates from verteporfin-treated cells (Fig. 6A–B). p62 immunoprecipitates from DMSO- and verteporfin-treated cells were immunoblotted for co-immunoprecipitated ubiquitinated proteins using an antibody that recognizes both mono- and polyubiquitinated protein chains [60]. In both MCF-7 EGFP-LC3 and BxPC-3 cells, exposure of cells to verteporfin increased the amount of polyubiquitinated proteins compared to control cells as detected by ubiquitin immunoblotting in the cell lysates prior to immunoprecipitation (Fig. 6A–B). Analysis of p62 immunoprecipitates for co-precipitated polyubiquitinated proteins clearly showed that a small fraction of cellular poly-ubiquinated proteins bind to p62 in untreated cells (Fig. 6A–B). However, despite the increase in polyubiquitinated proteins detected after verteporfin treatment, p62 immunoprecipitates from verteporfin-treated MCF-7 EGFP-LC3 cells contained less polyubiquitinated proteins than control cells (Fig. 6A). When the corresponding immunoprecipitate supernatants were probed for polyubiquitin, the supernatant from verteporfin-treated cells contained more polyubiquitinated proteins than found in the control supernatant (Fig. 6A), which most likely reflects both the increased cellular polyubiquitin content and its reduced binding to p62 caused by verteporfin. p62 immunoprecipitates from verteporfin-treated BxPC-3 cells appeared to contain amounts of co-immunoprecipitated polyubiquitinated proteins equal to DMSO-treated cells. However, keeping in mind that more p62 was present, and therefore immunoprecipitated, after exposure to verteporfin, equal amounts of co-immunoprecipitated polyubiquitin suggest there was relatively less ubiquitin bound to p62 after verteporfin treatment than beforehand (Fig. 6B). Due to unequal amounts of p62 between control- and verteporfin-treated immunoprecipitates, densitometric analysis was performed to quantify the entire co-immunoprecipitated polyubiquitin signal relative to the entire immunoprecipitated p62 signal before and after drug treatment (Fig. 6C). The ratio of polyubiquitin to p62 was markedly decreased after drug treatment in both MCF-7 EGFP-LC3 and BxPC-3 cell lines despite there being an overall increase in cellular polyubiquitinated content. The relative amount of polyubiquitin associated with p62 decreased by ∼50% and 85% after exposure to verteporfin in MCF-7 EGFP-LC3 and BxPC-3 cells, respectively (Fig. 6C). Therefore, generation of high-MW p62 by verteporfin interferes with its ability to associate with polyubiquitin, providing a possible mechanism for inhibition of autophagy by verteporfin.

To determine whether verteporfin treatment also interfered with LC3 binding to p62, we immunoprecipitated EGFP-LC3 from MCF-7 EGFP-LC3 cells treated with 10 µM verteporfin for 4 h and probed for p62. This experiment was carried out with the same lysates used in Fig. 6A. Analysis of the input after immunoprecipitation with GFP antibody showed complete isolation of EGFP-LC3 from the lysate (Fig. 6D). Co-immunoprecipitated p62 was strongly detected in EGFP-LC3 immunoprecipitates from both DMSO- and verteporfin-treated cells at 60 kDa; however, high-MW p62 forms were also detected in the EGFP-LC3 immunoprecipitate from verteporfin-treated cells, implying that high-MW p62 forms can still associate with LC3 (Fig. 6D). p62 western blot analysis of the lysate after EGFP-LC3 immunoprecipitation did not show any 60-kDa or high-MW p62 forms remaining, indicating that all endogenous p62 was bound to EGFP-LC3. This observation was surprising since p62 has many known binding partners, and it implies that LC3 and p62 are bound in the cytoplasm even under basal autophagy conditions in this EGFP-LC3 overexpressing cell line. GFP immunoprecipitation was also carried out in BxPC-3 cells, which do not express EGFP-LC3, to control for any nonspecific binding of p62 to the antibody or agarose protein G beads: no p62 was detected (Fig. 6D). These results demonstrate that both 60-kDa and high-MW p62 forms present after verteporfin treatment efficiently bind EGFP-LC3 despite the decreased association with polyubiquitinated proteins observed.

### PB1-mediated oligomerization is required for high-MW p62 formation by verteporfin

**Figure 7:**
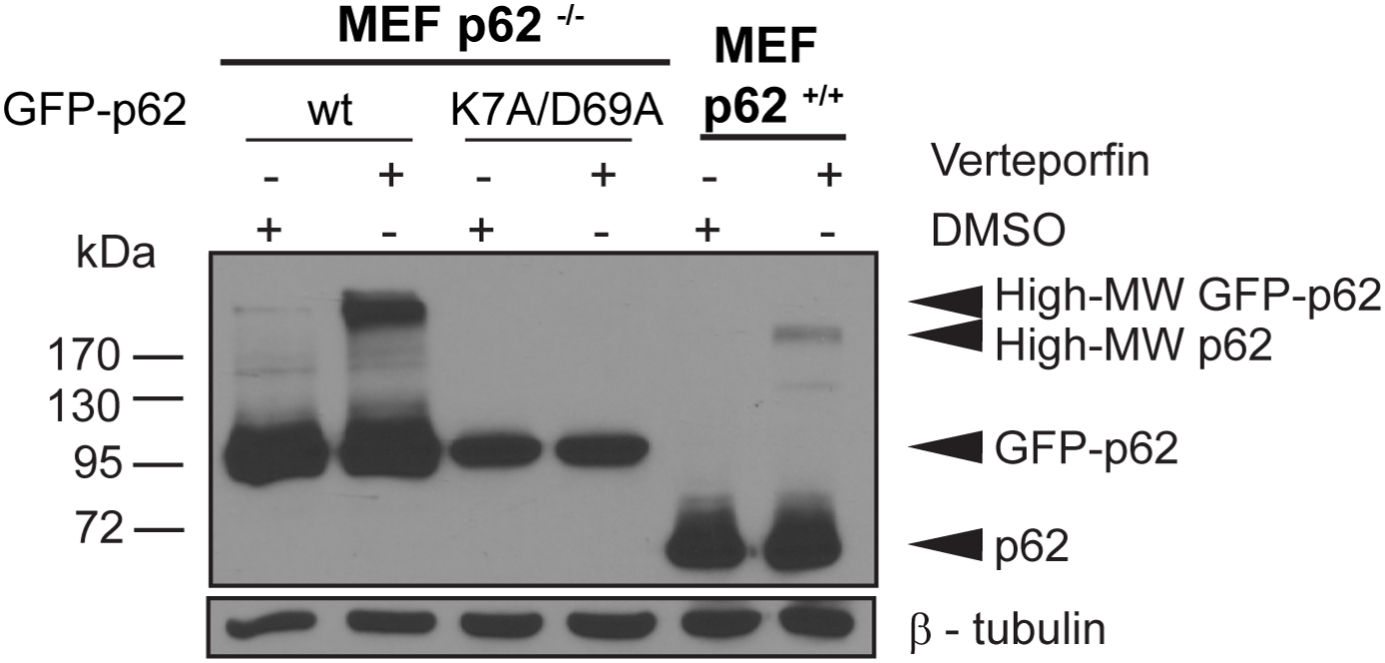
Effect of PB1 mutation on p62 crosslinking by verteporfin. p62^−/−^ MEF cells expressing GFP-p62 wt or GFP-p62 K7A/D69 or p62^+/+^ MEF cells were exposed to 0.1% DMSO or 10 µM verteporfin for 4 h in complete medium. Cell lysates were immunoblotted for p62 and β-tubulin. The image presented is representative of at least 3 independent experiments.

The N-terminal PB1 domain of p62 mediates its interaction with other proteins containing PB1 domains to form heterodimers and homo-oligomers [11], [12], [15]. The PB1 domain of p62 is unique in that it contains both an acidic and a basic interaction surface that enables p62 to self-oligomerize in a front-to-back orientation, thus forming homotypic arrays [11], [14], [15], a property that is critical for targeting p62 to the autophagosome formation site [61]. We tested whether a p62 PB1 mutant (K7A/D69A) that disrupts p62 self-oligomerization can form high-MW p62 in the presence of verteporfin. Using the Tet-On system, GFP-p62 K7A/D69A and GFP-p62 wt were expressed in p62^−/−^ MEF cells and p62 was monitored after 4 h treatment with verteporfin. High-MW GFP-p62 was observed only in the wild-type GFP-p62 expressing cells, and the amount of high-MW GFP-p62 increased significantly after verteporfin treatment (Fig. 7). By contrast, no high-MW p62 was detected in cells expressing the PB1 mutant, even after exposure to verteporfin (Fig. 7). As expected, verteporfin also generated high-MW endogenous p62 in p62^+/+^ MEF cells (Fig. 7). These results demonstrate that verteporfin-induced production of high-MW p62 crosslink products is dependent on p62 oligomerization through the PB1 domain. Therefore, it is likely that crosslinking amongst p62 monomers occurs via PB1 domain modification by verteporfin.

## Discussion

This study characterizes p62 as a cellular target of verteporfin, an inhibitor of autophagosome formation, and describes its modification through production of singlet oxygen by verteporfin. Verteporfin prevented autophagic degradation of p62 in autophagy-stimulating conditions, but it also generated high-MW forms of p62 stable to SDS denaturation and reducing conditions. Furthermore, verteporfin produced high-MW p62 *in vitro* from both p62 immunoprecipitates and purified recombinant GST-p62, demonstrating that the generation of high-MW p62 by verteporfin is a direct effect of verteporfin on p62 rather than a downstream consequence of autophagy inhibition, and that it can occur in the absence of any cellular constituents other than p62 itself. To date, the majority of verteporfin studies have focussed on cell killing in photodynamic therapy [6], [62]–[64], not on effects in the absence of light. In addition to its identification as an inhibitor of autophagosome formation, previous work from our group demonstrated that autophagy-inhibiting concentrations of verteporfin inhibit proliferation of some pancreatic cancer cell lines and induce high-MW p62 in cells and *in vivo*, all in the absence of photoactivation [5], [7]. It is notable that two recent publications showed non-photoactivated verteporfin also inhibits proliferation of hepatocellular carcinoma and retinoblastoma cell lines, perhaps through suppression of oncogenic YAP activity [65], [66]. *In vitro* experiments controlling for exposure to light and heat established that verteporfin was able to generate high-MW p62 from exposure to laboratory light at 4°C or in the absence of light at 37°C. The high-MW p62 SDS-PAGE migration patterns were similar irrespective of the energy source and resembled those observed after cell treatment. Thus, a photochemical mechanism underlies p62 modification by non-photoactivated verteporfin in purified p62 preparations and in cells.

In photodynamic therapy, red laser light excites verteporfin to a triplet state that initiates both primary and secondary photochemical reactions resulting in cytotoxic cellular damage. In biological environments, the light-activated triplet state of verteporfin transfers its energy to ground state oxygen, leading to the formation of singlet oxygen [6], [36], [37], [47], [67]. Rose bengal, another singlet oxygen producer, generated high-MW p62 in cells and *in vitro*, and excess histidine, an efficient singlet oxygen scavenger, decreased p62 modification, providing strong evidence that, even in the absence of light, singlet oxygen mediates p62 modification by verteporfin. As an electrophile, singlet oxygen readily reacts with and oxidizes the amino acids tryptophan, histidine, tyrosine, methionine, and cysteine [6], [37], [38]. Such oxidized residues are highly reactive towards other amino acid residues, resulting in protein crosslinking and the generation of more oxidized residues, many of which are characterized by carbonyl groups [37], [41], [43]. Accordingly, we detected an increase in the carbonyl content of purified recombinant His-p62 and GST-p62 in the high-MW region after exposure to either rose bengal or verteporfin in the dark, implying it is indeed a product of oxidative crosslinking. Singlet oxygen has an extremely short lifetime in the range of microseconds and a restricted diffusion range of 10–200 nm [68], [69] making crosslinking favorable among proteins that exist as oligomers, like p62 [47], [70]. Since high-MW p62 forms were observed at sizes ≥120 kDa and were not present in cells when p62 self-oligomerization was compromised, we conclude that high-MW forms of p62 are actually covalently crosslinked p62 oligomers.

Interestingly, in addition to our previous study [7], cellular high-MW p62 has been noted in three independent publications with little comment on its nature or consequences on cell function. Monick et al. (83) detected a p62 form with reduced SDS-PAGE mobility in alveolar macrophages obtained from long-term smokers while p62 was only found as the 60-kDa form in macrophages extracted from nonsmokers’ lungs. Cigarette smoke contains high levels of free radicals including nitric oxide, hydrogen peroxide, and singlet oxygen [71]–[74]. Zhao et al. [75] showed that exposure to UVA and UVB radiation generated high-MW p62 in keratinocytes that was not observed in unexposed cells. UV radiation is a known source of singlet oxygen [37], [76]. In these two papers, immunofluorescence microscopy showed p62 aggregation, leading the authors to speculate the high-MW p62 observed in western blots represented aggregated p62 rather than covalent crosslinking. However, protein aggregates are not expected to resist denaturation by SDS sample buffer and should migrate at their monomeric size. In our study, verteporfin generated high-MW p62 without causing p62 aggregation and induction of p62 aggregates did not generate SDS-stable high-MW p62. High-MW p62 has also been observed in aged cells in the absence of external singlet oxygen producers. Gamerdinger *et al.* [77] showed accumulation of high-MW p62, which they termed “SDS-stable SQSTM1 polymers,” in extracts from the cerebellum of aged mice that was not present in young cerebellum tissue, but the authors did not discuss the nature of this high-MW p62. It is interesting that high-MW p62 can also be observed in the absence of drug, in cellular and animal models of aging, particularly since it is well established that oxidized and crosslinked proteins accumulate in aged cells as a consequence of intracellular oxidative stress [77], [78]. Therefore, we reinterpret the above studies as evidence that exposure to cigarette smoke, UV light, or simply ageing can induce covalent p62 oligomers in cells. Furthermore, our data showing *in vitro* production of high-MW p62 in the presence of hydrogen peroxide and hypochlorous acid suggest ROS sources other than singlet oxygen may also contribute to p62 crosslinking in the models discussed.

To our knowledge, the effects of low-level singlet oxygen on autophagy have not been studied before. The extent of singlet oxygen generated by verteporfin without light activation is considerably lower than in photodynamic therapy conditions where ∼800-fold more energy is administered than under laboratory light [6], [47], [63]. In photodynamic therapy, photoactivated verteporfin elicits a rapid apoptotic response due to both mitochondrial photochemical damage and rapid accumulation of secondary ROS species [79]–[81]. By contrast, verteporfin is well characterized as being nontoxic to cells or humans in the absence of light irradiation [5], [6], [81] and it showed no toxicity towards cells under conditions used in this study. Like verteporfin, non-photoactivated rose bengal, a structurally distinct singlet oxygen producer, inhibited starvation-induced autophagosome accumulation in a manner that correlated with the appearance of p62 crosslink products. Itakura & Mizushima [61] showed that p62 oligomers associate with early autophagic structures at the autophagosome formation site. Due to the hydrophobic nature of verteporfin and the limited diffusion range of singlet oxygen, it is possible that verteporfin associates with the membrane of nascent autophagosomes to stimulate the crosslinking of p62 oligomers associated with these membranes.

**Figure 8:**
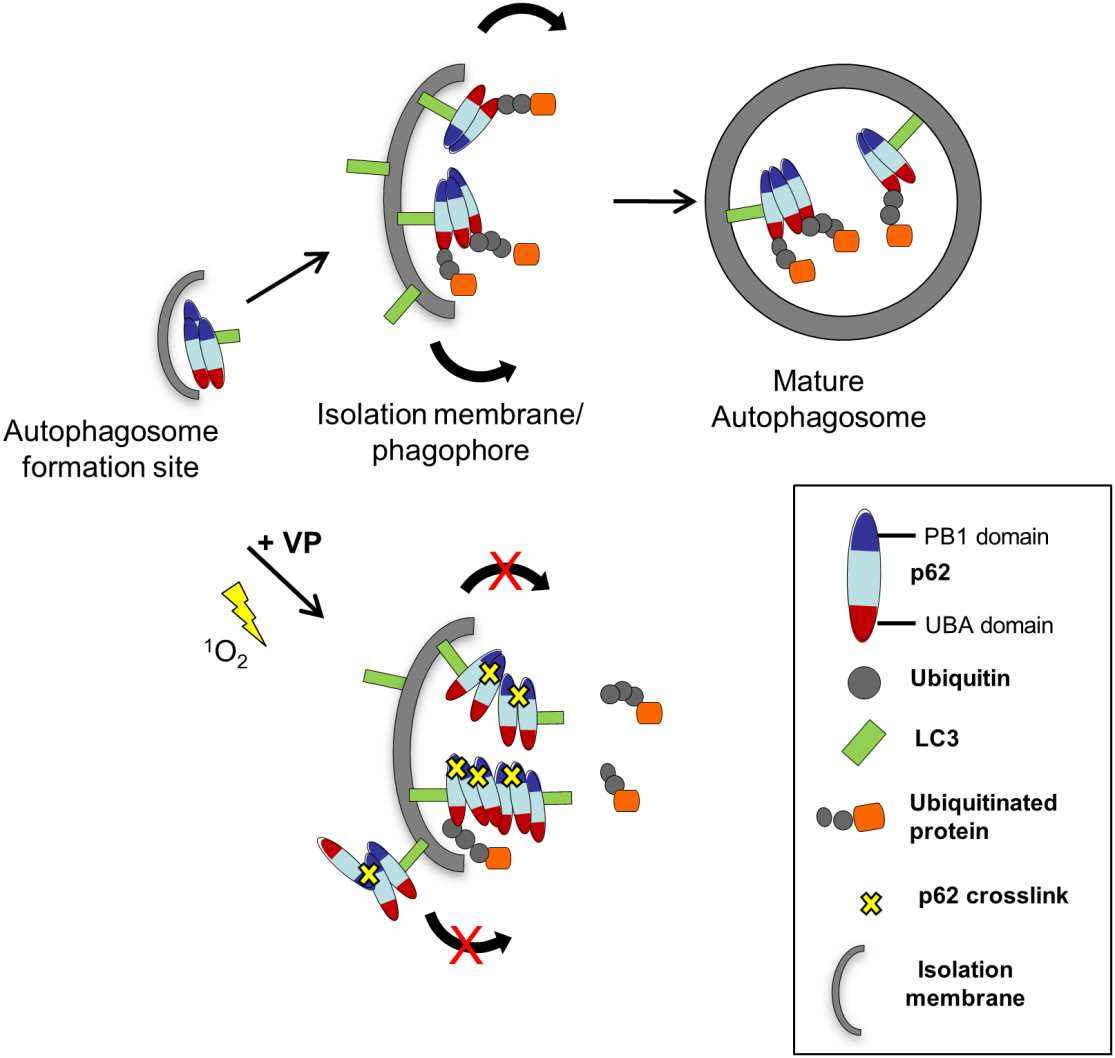
Proposed model for verteporfin-mediated inhibition of autophagosome formation involving p62 crosslink products. As p62 oligomers are recruited to the autophagosome membrane, they become oxidized and crosslinked to each other due to low-level singlet oxygen generation by verteporfin. This crosslinking event interferes with p62 binding to polyubiquitinated cargo, but does not affect LC3 binding. The generation of large p62 crosslink products with impaired function either physically disrupts proper autophagosome elongation and closure or it interferes with the function of other molecules necessary for completely autophagosome formation.

Protein oxidation and crosslinking can lead to loss of function depending on the site(s) and extent of modification [49], [51]. p62 immunoprecipitation showed verteporfin treatment compromised binding to polyubiquitinated proteins but not to LC3. p62 has been described as the major interacting protein of LC3 [10], and our data imply that p62 and LC3 are constitutively associated, at least in cells overexpressing EGFP-LC3 as used here. We have also shown that verteporfin does not affect LC3 lipidation or membrane association [5]. Based on these findings, we propose a model for verteporfin-mediated inhibition of autophagosome formation via p62 crosslinking (Fig. 8). In this model, p62 oligomers are initially recruited to the autophagosome formation site(s) by an LC3-independent mechanism [82]–[85]. LC3 bound to p62 oligomers is then recruited to the expanding phagophore membrane (Fig. 8). In the presence of verteporfin, p62 becomes crosslinked in a manner that disrupts its association with polyubiquitinated cargo, in agreement with observations that p62 binding to cargo is tightly regulated by both p62 oligomerization and dimerization of the UBA domain [61], [86], [87]. Regulation of expansion during autophagosome biogenesis is arguably the least understood subject in autophagy, but there is consensus in the field that it must be highly regulated to facilitate membrane curvature and eventual closure [88], [89]. We propose that abnormal products of p62 crosslinking prevent proper autophagosome formation through physical or mechanical disruption of the expanding membrane or by preventing the proper assembly of molecular factors responsible for autophagosome formation, perhaps through sequestration of LC3 into abnormal p62 oligomer crosslinks (Fig. 8). This hypothesis is consistent with previous electron microscopy data showing verteporfin treatment produced small single-membrane vesicles, presumably as a consequence of compromised autophagosome double-membrane expansion and closure [5]. Based on our data with rose bengal, we suspect that other non-photoactivated singlet oxygen generators may similarly inhibit autophagosome formation, depending on their subcellular localization and the amount of singlet oxygen produced.

High-MW p62 crosslinks were consistently observed in very low amounts in the absence of verteporfin, indicating that p62 might be particularly susceptible to exogenous and endogenous oxidants. Such susceptibility would also account for the accumulation of high-MW p62 observed in models of aging [77], [90]. Several studies have documented a decrease in autophagic proteolysis during aging [91]–[93]. It was also recently shown that overexpression of Atg 5 in transgenic mice activated autophagy and significantly extended their lifespan [93]–[95]. It is therefore hypothesized that compromised autophagy leads to increased intracellular accumulation of autophagic substrates in aged cells, such as damaged organelles and protein aggregates [90], [93], [96]. Based on our data, one plausible mechanism for autophagy impairment during aging is the accumulation of high-MW p62, during life-time exposure to low level oxidative stress.

By being highly susceptible to oxidation, p62 may perhaps also act as a physical sensor of oxidative stress. In addition to its function in autophagy, p62 plays an important role in activating cellular oxidative stress responses. p62 binds Keap1 through its KIR domain. In the absence of p62, Keap1 binds Nrf2, targeting Nrf2 for degradation via the ubiquitin-proteasome pathway [97], [98]. In the presence of oxidative and electrophilic stresses, Keap1 dissociates from Nrf2, likely though oxidation of key cysteine residues in Keap1, stabilizing Nrf2, thus allowing it to translocate into the nucleus where it activates transcription of a number of cytoprotective genes that contain antioxidant response elements, including NQO1 and p62 [97]–[99]. In addition to this canonical activation of Nrf2 signaling, phosphorylation of p62 during cellular stress has recently been found to enhance its binding to Keap1, sequestering Keap1 from Nrf2, thus participating in a feed-forward loop that activates and sustains the Keap1-Nrf2 oxidative stress response [8], [97], [100]–[104]. Furthermore, a recent study identified p62 as a target of lipid-derived electrophiles, which are products of oxidative stress that appear to serve as messengers by modulating cellular signaling [105]. Therefore, crosslinked high-MW p62 oligomers may be an early marker of oxidative stress and the consequences of their production in both physiological and pathophysiological contexts deserves further exploration.

## References

[1] Tanida I (2011) Autophagosome formation and molecular mechanism of autophagy. Antioxid Redox Signal 14:2201–2214. 20712405 10.1089/ars.2010.3482

[2] Levine B, Kroemer G (2009) Autophagy in aging, disease and death: the true identity of a cell death impostor. Cell Death Differ 16:1–2. 19079285 10.1038/cdd.2008.139 PMC2717606

[3] Balgi AD, Fonseca BD, Donohue E, Tsang TCF, Lajoie P, et al (2009) Screen for chemical modulators of autophagy reveals novel therapeutic inhibitors of mTORC1 signaling. PLoS One 4:e7124. 19771169 10.1371/journal.pone.0007124 PMC2742736

[4] Fleming A, Noda T, Yoshimori T, Rubinsztein DC (2011) Chemical modulators of autophagy as biological probes and potential therapeutics. Nat Chem Biol 7:9–17. 21164513 10.1038/nchembio.500

[5] Donohue E, Tovey A, Vogl AW, Arns S, Sternberg E, et al (2011) Inhibition of autophagosome formation by the benzoporphyrin derivative verteporfin. J Biol Chem 286:7290–7300. 21193398 10.1074/jbc.M110.139915 PMC3044985

[6] Schmidt-Erfurth U, Hasan T (2000) Mechanisms of action of photodynamic therapy with verteporfin for the treatment of age-related macular degeneration. Surv Ophthalmol 45:195–214. 11094244 10.1016/s0039-6257(00)00158-2

[7] Donohue E, Thomas A, Maurer N, Manisali I, Zeisser-Labouebe M, et al (2013) The autophagy inhibitor verteporfin moderately enhances the antitumor activity of gemcitabine in a pancreatic ductal adenocarcinoma model. J Cancer 4:585–596. 24069069 10.7150/jca.7030 PMC3781989

[8] Komatsu M, Ichimura Y (2010) Physiological significance of selective degradation of p62 by autophagy. FEBS Lett 584:1374–1378. 20153326 10.1016/j.febslet.2010.02.017

[9] Noda NN, Kumeta H, Nakatogawa H, Satoo K, Adachi W, et al (2008) Structural basis of target recognition by Atg8/LC3 during selective autophagy. Genes Cells 13:1211–1218. 19021777 10.1111/j.1365-2443.2008.01238.x

[10] Pankiv S, Clausen TH, Lamark T, Brech A, Bruun J-A, et al (2007) p62/SQSTM1 binds directly to Atg8/LC3 to facilitate degradation of ubiquitinated protein aggregates by autophagy. J Biol Chem 282:24131–24145. 17580304 10.1074/jbc.M702824200

[11] Lamark T, Perander M, Outzen H, Kristiansen K, Øvervatn A, et al (2003) Interaction codes within the family of mammalian Phox and Bem1p domain-containing proteins. J Biol Chem 278:34568–34581. 12813044 10.1074/jbc.M303221200

[12] Moscat J, Diaz-Meco MT, Albert A, Campuzano S (2006) Cell signaling and function organized by PB1 domain interactions. Mol Cell 23:631–640. 16949360 10.1016/j.molcel.2006.08.002

[13] Sumimoto H, Kamakura S, Ito T (2007) Structure and function of the PB1 domain, a protein interaction module conserved in animals, fungi, amoebas, and plants. Sci STKE 2007:re6. 17726178 10.1126/stke.4012007re6

[14] Ren J, Wang J, Wang Z, Wu J (2014) Structural and biochemical insights into the homotypic PB1-PB1 complex between PKCζ and p62. Sci China Life Sci 57:69–80. 24369353 10.1007/s11427-013-4592-z

[15] Wilson MI, Gill DJ, Perisic O, Quinn MT, Williams RL (2003) PB1 domain-mediated heterodimerization in NADPH oxidase and signaling complexes of atypical protein kinase C with Par6 and p62. Mol Cell 12:39–50. 12887891 10.1016/s1097-2765(03)00246-6

[16] Durán A, Serrano M, Leitges M, Flores JM, Picard S, et al (2004) The atypical PKC-interacting protein p62 is an important mediator of RANK-activated osteoclastogenesis. Dev Cell 6:303–309. 14960283 10.1016/s1534-5807(03)00403-9

[17] Ichimura Y, Kumanomidou T, Sou Y, Mizushima T, Ezaki J, et al (2008) Structural basis for sorting mechanism of p62 in selective autophagy. J Biol Chem 283:22847–22857. 18524774 10.1074/jbc.M802182200

[18] Men L, Wang Y (2007) The oxidation of yeast alcohol dehydrogenase-1 by hydrogen peroxide in vitro. J Proteome Res 6:216–225. 17203966 10.1021/pr0603809

[19] Lin H, Kenaan C, Zhang H, Hollenberg PF (2012) Reaction of human cytochrome P450 3A4 with peroxynitrite: nitrotyrosine formation on the proximal side impairs its interaction with NADPH-cytochrome P450 reductase. Chem Res Toxicol 25:2642–2653. 23016756 10.1021/tx3002753 PMC3524375

[20] Astier J, Besson-Bard A, Lamotte O, Bertoldo J, Bourque S, et al (2012) Nitric oxide inhibits the ATPase activity of the chaperone-like AAA+ ATPase CDC48, a target for S-nitrosylation in cryptogein signalling in tobacco cells. Biochem J 447:249–260. 22835150 10.1042/BJ20120257

[21] Klionsky DDJ, Abdalla FFC, Abeliovich H, Abarham RT, Abraham RT, et al (2012) Guidelines for the use and interpretation of assays for monitoring autophagy. Autophagy 8:445–544. 22966490 10.4161/auto.19496 PMC3404883

[22] Ichimura Y, Kominami E, Tanaka K, Komatsu M (2008) Selective turnover of p62/A170/SQSTM1 by autophagy. Autophagy 4:1063–1066. 18776737 10.4161/auto.6826

[23] Bjørkøy G, Lamark T, Brech A, Outzen H, Perander M, et al (2005) p62/SQSTM1 forms protein aggregates degraded by autophagy and has a protective effect on huntingtin-induced cell death. J Cell Biol 171:603–614. 16286508 10.1083/jcb.200507002 PMC2171557

[24] Zatloukal K, Stumptner C, Fuchsbichler A, Heid H, Schnoelzer M, et al (2002) p62 Is a common component of cytoplasmic inclusions in protein aggregation diseases. Am J Pathol 160:255–263. 11786419 10.1016/S0002-9440(10)64369-6 PMC1867135

[25] Kuusisto E, Salminen A, Alafuzoff I (2001) Ubiquitin-binding protein p62 is present in neuronal and glial inclusions in human tauopathies and synucleinopathies. Neuroreport 12:2085–2090. 11447312 10.1097/00001756-200107200-00009

[26] Nagaoka U, Kim K, Jana NR, Doi H, Maruyama M, et al (2004) Increased expression of p62 in expanded polyglutamine-expressing cells and its association with polyglutamine inclusions. J Neurochem 91:57–68. 15379887 10.1111/j.1471-4159.2004.02692.x

[27] Ravikumar B, Rubinsztein DC (2004) Can autophagy protect against neurodegeneration caused by aggregate-prone proteins? Neuroreport 15:2443–2445. 15538170 10.1097/00001756-200411150-00001

[28] Kamimoto T, Shoji S, Hidvegi T, Mizushima N, Umebayashi K, et al (2006) Intracellular inclusions containing mutant alpha1-antitrypsin Z are propagated in the absence of autophagic activity. J Biol Chem 281:4467–4476. 16365039 10.1074/jbc.M509409200

[29] Komatsu M, Waguri S, Koike M, Sou Y-S, Ueno T, et al (2007) Homeostatic levels of p62 control cytoplasmic inclusion body formation in autophagy-deficient mice. Cell 131:1149–1163. 18083104 10.1016/j.cell.2007.10.035

[30] Komatsu M, Waguri S, Chiba T, Murata S, Iwata J, et al (2006) Loss of autophagy in the central nervous system causes neurodegeneration in mice. Nature 441:880–884. 16625205 10.1038/nature04723

[31] Hara T, Nakamura K, Matsui M, Yamamoto A, Nakahara Y, et al (2006) Suppression of basal autophagy in neural cells causes neurodegenerative disease in mice. Nature 441:885–889. 16625204 10.1038/nature04724

[32] Hunt DW, Jiang HJ, Levy JG, Chan a H (1995) Sensitivity of activated murine peritoneal macrophages to photodynamic killing with benzoporphyrin derivative. Photochem Photobiol 61:417–421. 7740088 10.1111/j.1751-1097.1995.tb08633.x

[33] Renno RZ, Terada Y, Haddadin MJ, Michaud N a, Gragoudas ES, et al (2004) Selective photodynamic therapy by targeted verteporfin delivery to experimental choroidal neovascularization mediated by a homing peptide to vascular endothelial growth factor receptor-2. Arch Ophthalmol 122:1002–1011. 15249365 10.1001/archopht.122.7.1002

[34] Levy JG (1995) Photodynamic therapy. Trends Biotechnol 13:14–18. 7765800 10.1016/S0167-7799(00)88895-2

[35] Foote CS, Shook FC, Abakerli RB (1984) Characterization of singlet oxygen. Methods Enzymol 105:36–47. 6727677 10.1016/s0076-6879(84)05006-0

[36] DeRosa MC, Crutchley RJ (2002) Photosensitized singlet oxygen and its applications. Coord Chem Rev 233–234:351–371.

[37] Pattison DI, Rahmanto AS, Davies MJ (2012) Photo-oxidation of proteins. Photochem Photobiol Sci 11:38–53. 21858349 10.1039/c1pp05164d

[38] Davies MJ (2003) Singlet oxygen-mediated damage to proteins and its consequences. Biochem Biophys Res Commun 305:761–770. 12763058 10.1016/s0006-291x(03)00817-9

[39] Davies MJ (2004) Reactive species formed on proteins exposed to singlet oxygen. Photochem Photobiol Sci 3:17–25. 14743273 10.1039/b307576c

[40] Giulivi C, Traaseth NJ, Davies KJ a (2003) Tyrosine oxidation products: analysis and biological relevance. Amino Acids 25:227–232. 14661086 10.1007/s00726-003-0013-0

[41] Shen HR, Spikes JD, Kopecková P, Kopecek J (1996) Photodynamic crosslinking of proteins. II. Photocrosslinking of a model protein-ribonuclease A. J Photochem Photobiol B 35:213–219. 8933727 10.1016/s1011-1344(96)07300-9

[42] Verweu H, Steveninck J van (1982) Model studies on photodynamic cross-linking. Photochem Photobiol 35:265–267.

[43] Shen HR, Spikes JD, Kopeceková P, Kopecek J (1996) Photodynamic crosslinking of proteins. I. Model studies using histidine- and lysine-containing N-(2-hydroxypropyl)methacrylamide copolymers. J Photochem Photobiol B 34:203–210. 8810538 10.1016/1011-1344(96)07286-7

[44] Dalsgaard TK, Otzen D, Nielsen JH, Larsen LB (2007) Changes in structures of milk proteins upon photo-oxidation. J Agric Food Chem 55:10968–10976. 18044829 10.1021/jf071948g

[45] Goosey JD, Zigler JS, Kinoshita JH (1980) Cross-linking of lens crystallins in a photodynamic system: a process mediated by singlet oxygen. Science 208:1278–1280. 7375939 10.1126/science.7375939

[46] Henderson BW, Daroqui C, Tracy E, Vaughan LA, Loewen GM, et al (2007) Cross-linking of signal transducer and activator of transcription 3–a molecular marker for the photodynamic reaction in cells and tumors. Clin Cancer Res 13:3156–3163. 17545518 10.1158/1078-0432.CCR-06-2950

[47] Bae SI, Zhao R, Snapka RM (2008) PCNA damage caused by antineoplastic drugs. Biochem Pharmacol 76:1653–1668. 18823950 10.1016/j.bcp.2008.09.003 PMC2659951

[48] Paczkowski J, Lamberts JJ, Paczkowska B, Neckers DC (1985) Photophysical properties of rose bengal and its derivatives (XII). J Free Radic Biol Med 1:341–351. 3837801 10.1016/0748-5514(85)90146-1

[49] Luo J, Li L, Zhang Y, Spitz DR, Buettner GR, et al (2006) Inactivation of primary antioxidant enzymes in mouse keratinocytes by photodynamically generated singlet oxygen. Antioxid Redox Signal 8:1307–1314. 16910778 10.1089/ars.2006.8.1307

[50] Agon V V, Bubb W a, Wright A, Hawkins CL, Davies MJ (2006) Sensitizer-mediated photooxidation of histidine residues: evidence for the formation of reactive side-chain peroxides. Free Radic Biol Med 40:698–710 doi:10.1016/j.freeradbiomed.2005.09.039 16458201 10.1016/j.freeradbiomed.2005.09.039

[51] Kim SY, Kwon OJ, Park JW (2001) Inactivation of catalase and superoxide dismutase by singlet oxygen derived from photoactivated dye. Biochimie 83:437–444. 11368853 10.1016/s0300-9084(01)01258-5

[52] Gornati R, Colombo G, Clerici M, Rossi F, Gagliano N, et al (2013) Protein carbonylation in human endothelial cells exposed to cigarette smoke extract. Toxicol Lett 218:118–128. 23396223 10.1016/j.toxlet.2013.01.023

[53] Suto D, Iuchi Y, Ikeda Y, Sato K, Ohba Y, et al (2007) Inactivation of cysteine and serine proteases by singlet oxygen. Arch Biochem Biophys 461:151–158. 17459324 10.1016/j.abb.2007.03.020

[54] Suzuki YJ, Carini M, Butterfield DA (2010) Protein carbonylation. Antioxid Redox Signal 12:323–325. 19743917 10.1089/ars.2009.2887 PMC2821144

[55] Dalle-Donne I, Rossi R, Giustarini D, Milzani A, Colombo R (2003) Protein carbonyl groups as biomarkers of oxidative stress. Clin Chim Acta 329:23–38. 12589963 10.1016/s0009-8981(03)00003-2

[56] Tomita M, Irie M, Ukita T (1969) Sensitized photooxidation of histidine and its derivatives. Products and mechanism of the reaction. Biochemistry 8:5149–5160. 5365801 10.1021/bi00840a069

[57] Pacher P, Beckman JS, Liaudet L (2007) Nitric oxide and peroxynitrite in health and disease. Physiol Rev 87:315–424. 17237348 10.1152/physrev.00029.2006 PMC2248324

[58] Alarcón E, Edwards AM, Aspée A, Borsarelli CD, Lissi EA (2009) Photophysics and photochemistry of rose bengal bound to human serum albumin. Photochem Photobiol Sci 8:933–943. 19582268 10.1039/b901056d

[59] He Y-Y, Council SE, Feng L, Bonini MG, Chignell CF (2008) Spatial distribution of protein damage by singlet oxygen in keratinocytes. Photochem Photobiol 84:69–74. 18173704 10.1111/j.1751-1097.2007.00199.x PMC2365760

[60] Geisler S, Holmström KM, Skujat D, Fiesel FC, Rothfuss OC, et al (2010) PINK1/Parkin-mediated mitophagy is dependent on VDAC1 and p62/SQSTM1. Nat Cell Biol 12:119–131. 20098416 10.1038/ncb2012

[61] Itakura E, Mizushima N (2011) p62 Targeting to the autophagosome formation site requires self-oligomerization but not LC3 binding. J Cell Biol 192:17–27. 21220506 10.1083/jcb.201009067 PMC3019556

[62] Pogue BW, O’Hara JA, Demidenko E, Wilmot CM, Goodwin IA, et al (2003) Photodynamic therapy with verteporfin in the radiation-induced fibrosarcoma-1 tumor causes enhanced radiation sensitivity. Cancer Res 63:1025–1033. 12615718

[63] Chen B, Pogue BW, Hoopes PJ, Hasan T (2005) Combining vascular and cellular targeting regimens enhances the efficacy of photodynamic therapy. Int J Radiat Oncol Biol Phys 61:1216–1226. 15752904 10.1016/j.ijrobp.2004.08.006

[64] Fateye B, Li W, Wang C, Chen B (2012) Combination of phosphatidylinositol 3-kinases pathway inhibitor and photodynamic therapy in endothelial and tumor cells. Photochem Photobiol 88:1265–1272. 22506666 10.1111/j.1751-1097.2012.01160.x

[65] Brodowska K, Al-Moujahed A, Marmalidou A, Meyer Zu Horste M, Cichy J, et al (2014) The clinically used photosensitizer Verteporfin (VP) inhibits YAP-TEAD and human retinoblastoma cell growth in vitro without light activation. Exp Eye Res 124:67–73. 24837142 10.1016/j.exer.2014.04.011 PMC4135181

[66] Liu-Chittenden Y, Huang B, Shim JS, Chen Q, Lee S-J, et al (2012) Genetic and pharmacological disruption of the TEAD-YAP complex suppresses the oncogenic activity of YAP. Genes Dev 26:1300–1305. 22677547 10.1101/gad.192856.112 PMC3387657

[67] Min DB, Boff JM (2002) Chemistry and Reaction of Singlet Oxygen in Foods. Compr Rev Food Sci Food Saf 1:58–72. 10.1111/j.1541-4337.2002.tb00007.x 33451241

[68] Moan J, Berg K (1991) The photodegradation of porphyrins in cells can be used to estimate the lifetime of singlet oxygen. Photochem Photobiol 53:549–553. 1830395 10.1111/j.1751-1097.1991.tb03669.x

[69] Redmond RW, Kochevar IE (2006) Spatially resolved cellular responses to singlet oxygen. Photochem Photobiol 82:1178–1186. 16740059 10.1562/2006-04-14-IR-874

[70] Ferrario A, Rucker N, Wong S, Luna M, Gomer CJ (2007) Survivin, a member of the inhibitor of apoptosis family, is induced by photodynamic therapy and is a target for improving treatment response. Cancer Res 67:4989–4995. 17510430 10.1158/0008-5472.CAN-06-4785

[71] Lyons MJ, Gibson JF, Ingram DJ (1958) Free-radicals produced in cigarette smoke. Nature 181:1003–1004. 13541350 10.1038/1811003a0

[72] Epperlein MM, Nourooz-Zadeh J, Noronha-Dutra AA, Woolf N (1996) Nitric oxide in cigarette smoke as a mediator of oxidative damage. Int J Exp Pathol 77:197–200. 8977370 10.1046/j.1365-2613.1996.9930331.x PMC2691640

[73] Michael Pittilo R (2000) Cigarette smoking, endothelial injury and cardiovascular disease. Int J Exp Pathol 81:219–230. 10971743 10.1046/j.1365-2613.2000.00162.x PMC2517732

[74] Salem AF, Al-Zoubi MS, Whitaker-Menezes D, Martinez-Outschoorn UE, Lamb R, et al (2013) Cigarette smoke metabolically promotes cancer, via autophagy and premature aging in the host stromal microenvironment. Cell Cycle 12:818–825. 23388463 10.4161/cc.23722 PMC3610729

[75] Zhao Y, Zhang C-F, Rossiter H, Eckhart L, König U, et al. (2013) Autophagy Is Induced by UVA and Promotes Removal of Oxidized Phospholipids and Protein Aggregates in Epidermal Keratinocytes. J Invest Dermatol. 10.1038/jid.2013.26 23340736

[76] Berneburg M, Grether-Beck S, Kürten V, Ruzicka T, Briviba K, et al (1999) Singlet oxygen mediates the UVA-induced generation of the photoaging-associated mitochondrial common deletion. J Biol Chem 274:15345–15349. 10336420 10.1074/jbc.274.22.15345

[77] Gamerdinger M, Hajieva P, Kaya AM, Wolfrum U, Hartl FU, et al (2009) Protein quality control during aging involves recruitment of the macroautophagy pathway by BAG3. EMBO J 28:889–901. 19229298 10.1038/emboj.2009.29 PMC2647772

[78] Berlett BS (1997) Protein Oxidation in Aging, Disease, and Oxidative Stress. J Biol Chem 272:20313–20316. 9252331 10.1074/jbc.272.33.20313

[79] Granville DJ, Hunt DW (2000) Porphyrin-mediated photosensitization - taking the apoptosis fast lane. Curr Opin Drug Discov Devel 3:232–243. 19649854

[80] Kessel D, Vicente MGH, Reiners JJ (2006) Initiation of apoptosis and autophagy by photodynamic therapy. Autophagy 2:289–290. 16921269 10.4161/auto.2792 PMC2747798

[81] Granville D, Shaw J, Leong S (1999) Release of Cytochrome c, Bax Migration, Bid Cleavage, and Activation of Caspases 2, 3, 6, 7, 8, and 9 during Endothelial Cell Apoptosis. Am J Pathol Pathol 155:1021–1025. 10.1016/S0002-9440(10)65202-9 PMC1867016 10514382

[82] Hamasaki M, Furuta N, Matsuda A, Nezu A, Yamamoto A, et al (2013) Autophagosomes form at ER–mitochondria contact sites. Nature 495:389–393. 23455425 10.1038/nature11910

[83] Hailey DW, Rambold AS, Satpute-Krishnan P, Mitra K, Sougrat R, et al (2010) Mitochondria supply membranes for autophagosome biogenesis during starvation. Cell 141:656–667. 20478256 10.1016/j.cell.2010.04.009 PMC3059894

[84] Hayashi-Nishino M, Fujita N, Noda T, Yamaguchi A, Yoshimori T, et al (2009) A subdomain of the endoplasmic reticulum forms a cradle for autophagosome formation. Nat Cell Biol 11:1433–1437. 19898463 10.1038/ncb1991

[85] Itakura E, Mizushima N (2010) Characterization of autophagosome formation site by a hierarchical analysis of mammalian Atg proteins. Autophagy 6:764–776. 20639694 10.4161/auto.6.6.12709 PMC3321844

[86] Isogai S, Morimoto D, Arita K, Unzai S, Tenno T, et al (2011) Crystal structure of the ubiquitin-associated (UBA) domain of p62 and its interaction with ubiquitin. J Biol Chem 286:31864–31874. 21715324 10.1074/jbc.M111.259630 PMC3173063

[87] Saitoh T, Fujita N, Jang MH, Uematsu S, Yang B-G, et al (2008) Loss of the autophagy protein Atg16L1 enhances endotoxin-induced IL-1beta production. Nature 456:264–268. 18849965 10.1038/nature07383

[88] Webber JL, Tooze SA (2010) New insights into the function of Atg9. FEBS Lett 584:1319–1326. 20083107 10.1016/j.febslet.2010.01.020

[89] Eskelinen E-L (2005) Maturation of autophagic vacuoles in Mammalian cells. Autophagy 1:1–10. 16874026 10.4161/auto.1.1.1270

[90] Bitto A, Lerner CA, Nacarelli T, Crowe E, Torres C, et al. (2014) p62/SQSTM1 at the interface of aging, autophagy, and disease. Age (Dordr). 10.1007/s11357-014-9626-3 PMC4082582 24557832

[91] Del Roso A, Vittorini S, Cavallini G, Donati A, Gori Z, et al (2003) Ageing-related changes in the in vivo function of rat liver macroautophagy and proteolysis. Exp Gerontol 38:519–527. 12742529 10.1016/s0531-5565(03)00002-0

[92] Donati A, Cavallini G, Paradiso C, Vittorini S, Pollera M, et al (2001) Age-related changes in the autophagic proteolysis of rat isolated liver cells: effects of antiaging dietary restrictions. J Gerontol A Biol Sci Med Sci 56:B375–83. 11524438 10.1093/gerona/56.9.b375

[93] Cuervo AM, Bergamini E, Brunk UT, Dröge W, Ffrench M, et al (2005) Autophagy and aging: the importance of maintaining “clean” cells. Autophagy 1:131–140. 16874025 10.4161/auto.1.3.2017

[94] Pyo J-O, Yoo S-M, Ahn H-H, Nah J, Hong S-H, et al (2013) Overexpression of Atg5 in mice activates autophagy and extends lifespan. Nat Commun 4:2300. 23939249 10.1038/ncomms3300 PMC3753544

[95] Vittorini S, Paradiso C, Donati A, Cavallini G, Masini M, et al (1999) The age-related accumulation of protein carbonyl in rat liver correlates with the age-related decline in liver proteolytic activities. J Gerontol A Biol Sci Med Sci 54:B318–23. 10496537 10.1093/gerona/54.8.b318

[96] Pyo J-O, Yoo S-M, Jung Y-K (2013) The Interplay between Autophagy and Aging. Diabetes Metab J 37:333–339. 24199161 10.4093/dmj.2013.37.5.333 PMC3816133

[97] Taguchi K, Fujikawa N, Komatsu M, Ishii T, Unno M, et al (2012) Keap1 degradation by autophagy for the maintenance of redox homeostasis. Proc Natl Acad Sci U S A 109:13561–13566. 22872865 10.1073/pnas.1121572109 PMC3427110

[98] Komatsu M, Kurokawa H, Waguri S, Taguchi K, Kobayashi A, et al (2010) The selective autophagy substrate p62 activates the stress responsive transcription factor Nrf2 through inactivation of Keap1. Nat Cell Biol 12:213–223. 20173742 10.1038/ncb2021

[99] Hayes JD, McMahon M (2009) NRF2 and KEAP1 mutations: permanent activation of an adaptive response in cancer. Trends Biochem Sci 34:176–188. 19321346 10.1016/j.tibs.2008.12.008

[100] Komatsu M, Kageyama S, Ichimura Y (2012) p62/SQSTM1/A170: physiology and pathology. Pharmacol Res 66:457–462. 22841931 10.1016/j.phrs.2012.07.004

[101] Inami Y, Waguri S, Sakamoto A, Kouno T, Nakada K, et al (2011) Persistent activation of Nrf2 through p62 in hepatocellular carcinoma cells. J Cell Biol 193:275–284. 21482715 10.1083/jcb.201102031 PMC3080263

[102] Ichimura Y, Waguri S, Sou Y-S, Kageyama S, Hasegawa J, et al (2013) Phosphorylation of p62 activates the Keap1-Nrf2 pathway during selective autophagy. Mol Cell 51:618–631. 24011591 10.1016/j.molcel.2013.08.003

[103] Lau A, Wang X-J, Zhao F, Villeneuve NF, Wu T, et al (2010) A noncanonical mechanism of Nrf2 activation by autophagy deficiency: direct interaction between Keap1 and p62. Mol Cell Biol 30:3275–3285. 20421418 10.1128/MCB.00248-10 PMC2897585

[104] Copple IM, Lister A, Obeng AD, Kitteringham NR, Jenkins RE, et al (2010) Physical and functional interaction of sequestosome 1 with Keap1 regulates the Keap1-Nrf2 cell defense pathway. J Biol Chem 285:16782–16788. 20378532 10.1074/jbc.M109.096545 PMC2878012

[105] Wang C, Weerapana E, Blewett MM, Cravatt BF (2014) A chemoproteomic platform to quantitatively map targets of lipid-derived electrophiles. Nat Methods 11:79–85. 24292485 10.1038/nmeth.2759 PMC3901407

